# A compendium of long non-coding RNAs transcriptional fingerprint in multiple myeloma

**DOI:** 10.1038/s41598-018-24701-8

**Published:** 2018-04-26

**Authors:** Domenica Ronchetti, Luca Agnelli, Alessandro Pietrelli, Katia Todoerti, Martina Manzoni, Elisa Taiana, Antonino Neri

**Affiliations:** 1 Department of Oncology and Hemato-oncology, University of Milan, Milan, Italy; 2 Hematology, Fondazione Cà Granda IRCCS Policlinico, Milan, Italy; 3 Internal Medicine and Metabolic Diseases, Fondazione IRCCS Ca’ Granda Ospedale Policlinico, Milan, Italy; 4 Bioinformatic Unit, Istituto Nazionale Genetica Molecolare, Milan, Italy

## Abstract

Multiple myeloma (MM) is a clonal proliferation of bone marrow plasma cells characterized by highly heterogeneous genetic background and clinical course, whose pathogenesis remains largely unknown. Long ncRNAs (lncRNAs) are a large class of non-protein-coding RNA, involved in many physiological cellular and genomic processes as well as in carcinogenesis and tumor evolution. Although still in its infancy, the role of lncRNAs in MM is progressively expanding. Besides studies on selected candidates, lncRNAs expression at genome-wide transcriptome level is confined to microarray technologies, thus investigating a limited collection of transcripts. In the present study investigating a cohort of 30 MM patients, a deep RNA-sequencing analysis overwhelmed previous array studies and allowed the most accurate definition of lncRNA transcripts structure and expression, ultimately providing a comprehensive catalogue of lncRNAs specifically associated with the main MM molecular subgroups and genetic alterations. Despite the small number of analyzed samples, the high accuracy of RNA-sequencing approach for complex transcriptome processing led to the identification of 391 deregulated lncRNAs, 67% of which were also detectable and validated by whole-transcript microarrays. In addition, we identified a list of lncRNAs, with potential relevance in MM, co-expressed and in close proximity to genes that might undergo a cis-regulatory relationship.

## Introduction

Multiple myeloma (MM) is an uncontrolled proliferation of Ig-secreting plasma cells (PCs) that accounts for 10% of all hematological tumors with incidence in Western countries of about 3–5 per 100,000. Despite the extraordinary progresses in the diagnosis and treatment of the disease^1^, MM remains still incurable.

At the genetic level, MM is characterized by both numerical and structural chromosomal alterations, i.e. translocations affecting immunoglobulin heavy chain (IGH) locus and a number of oncogenic partners, hyperdiploidy (HD), deletions of 13q and 17p13, and gain of 1q^2^. In addition, whole genome/exome sequencing analyses recently evidenced somatic mutations occurring in genes with putative pathogenetic role, such *KRAS*, *NRAS*, *TP53*, *BRAF*, *TRAF3*, *FAM46C* and *DIS3*^3,4^.

To date, many efforts have been undertaken to investigate the different molecular types of MM, aimed at understanding the clinical heterogeneity of the disease and promptly/easily identifying patients more prone to disease progression or relapse. Starting from global coding-gene expression profiling of purified myeloma PCs, it became possible to identify peculiar tumor-associated transcriptional profiles discriminating between normal and tumor phenotypes, or specifically associated with distinct MM molecular subtypes with different prognosis^5,6^. Afterwards, the increasing discovery of non-coding RNAs (ncRNAs), following human genome sequencing, have been changing the landscape of cancer biology. Focusing on MM, the investigations on small nucleolar RNA and mostly microRNAs (miRNAs), have greatly contributed to shed light into the molecular mechanism of the pathology and provide some new potential molecular targets^7–10^.

More recently, great attention has been dedicated to the heterogeneous group of long non-coding RNAs (lncRNAs). Genome-wide transcriptional studies carried out by ENCODE (Encyclopedia of DNA Elements) and other large international consortia have revealed that more than 90% of mammalian genomes is transcribed and that a great part of the transcripts are lncRNAs^11–13^. The GENCODE consortium^14^ has arranged a comprehensive set of human lncRNAs and analyzed their genomic organization, modifications, cellular localizations and tissue expression profiles in different human cell line. LncRNAs contribute to several processes, e.g. maintenance of genomic integrity, X-chromosome inactivation, transcriptional regulation, genomic imprinting, cell differentiation and development^15,16^. Several lncRNAs have also been described to contribute to tumor formation and/or progression, as well as to metastatic processes, in many solid and hematologic tumors^17,18^, showing either oncogenic or tumor suppressive function.

Although the investigation of lncRNA in MM is still in its beginnings, our understanding of their role is progressively expanding. One of the most investigated lncRNA is MALAT1, deregulated in many solid tumors with a putatively oncogenic function^19,20^. MALAT1 is overexpressed in MM, where it has been shown to predict tumor progression^21^. Recent researches in MM have been focused on single lncRNAs already known as involved in different types of cancers, such as MEG3 functioning as tumor suppressor through both p53-dependent and p53-independent mechanisms^22^, or CRNDE overexpressed in association with poor clinical outcome^23^. Besides studies on selected candidates, lncRNA expression at genome-wide transcriptome level has been scarcely investigated in MM and the only two efforts reported so far are based on microarray data. In particular, Zhou *et al*.^24^ investigated a repertoire of 2,330 lncRNAs in a publicly available clinically annotated cohort of 559 MM patients generating a four-lncRNA prognostic signature. In a previous study, our group analyzed the transcriptional patterns of 1,852 lncRNAs in 259 patients affected by the different forms of PC dyscrasia at onset, included in proprietary and publicly available datasets, identifying a series of deregulated lncRNAs associated either with disease progression or distinct molecular subgroups of MM^25^. However, these studies are limited to the detection of a relatively small number of sequences queried by the arrays, which were primarily designed to detect the coding transcriptome. Next-generation RNA sequencing (RNA-seq) addresses this shortcoming, but to date such an approach has not yet been pursued in MM.

In the present study, we investigated the lncRNA expression profiling in MM patients by RNA-seq, with the aim of providing a first exhaustive catalogue of lncRNAs specifically associated with the main molecular subgroups and genetic alterations in MM. Furthermore, we defined a repertoire of lncRNAs possibly involved in MM, as they meet the requirements of being both co-expressed and in close proximity to genes that have been described as relevant to this neoplasia, thus suggestive of a cis-regulatory relationship.

Overall, such a compendium and the free availability of RNA-seq data may provide the scientific community with valuable references for future research into the involvement of lncRNAs in MM.

## Results

### LncRNAs expression profile in multiple myeloma

The expression profile of lncRNAs has been investigated by RNA-seq in a cohort of 30 MM patients at diagnosis, whose molecular features were representative of those mainly characterizing the disease (Table 1).

**Table 1:** Molecular characteristics of 30 MM patients.

Sample Features	Positive (%)	Negative (%)	Not available
HD	8 (27)	20 (67)	2
t(11;14)	8 (27)	22 (73)	0
t(4;14)	7 (23)	23 (77)	0
MAF-trx	4 (13)	26 (87)	0
del(17)	3 (10)	27 (90)	0
del(13)	18 (60)	12 (40)	0
1q-gain	15 (50)	13 (43)	2
*N-RAS*	3 (10)	20 (67)	7
*K-RAS*	7 (23)	16 (53)	7
*BRAF*	4 (13)	19 (63)	7
*DIS3*	6 (20)	17 (57)	7
*P53*	2(6)	20(67)	8
*FAM46C*	1(3)	21(70)	8

We used a custom pipeline, based on the GENCODE encyclopedia that considered only those genes with unambiguously mapped transcripts, that allowed to annotate 14,202 lncRNAs; among them, we investigated the 9,540 lncRNAs detectable upon removal of those unexpressed across the whole dataset. Overall, lncRNAs are scarcely expressed. Indeed, for each lncRNA the sum of the read counts in the 30 samples spans a wide range of values (from 2 to 6,707,843; median: 57). However, 86% of the 9,540 lncRNAs have average read counts <30, whereas only 1% of lncRNAs show average values > 500. Notably, 12 lncRNAs are very highly expressed displaying an average read counts >5000, counting 64% of the reads assigned to lncRNAs (Supplementary Table S1); in particular, this group includes *NEAT*1, *MALAT*1, *MIAT* and *TUG1* frequently deregulated in malignant B-cells^26^. Based on the rationale that a single cis-acting molecule might be able to target effectively a neighboring locus, thus suggesting that even low expressed lncRNAs may have a key regulatory role^27^, we considered all the 9,540 detectable lncRNAs for subsequent investigations.

**Figure 1:**
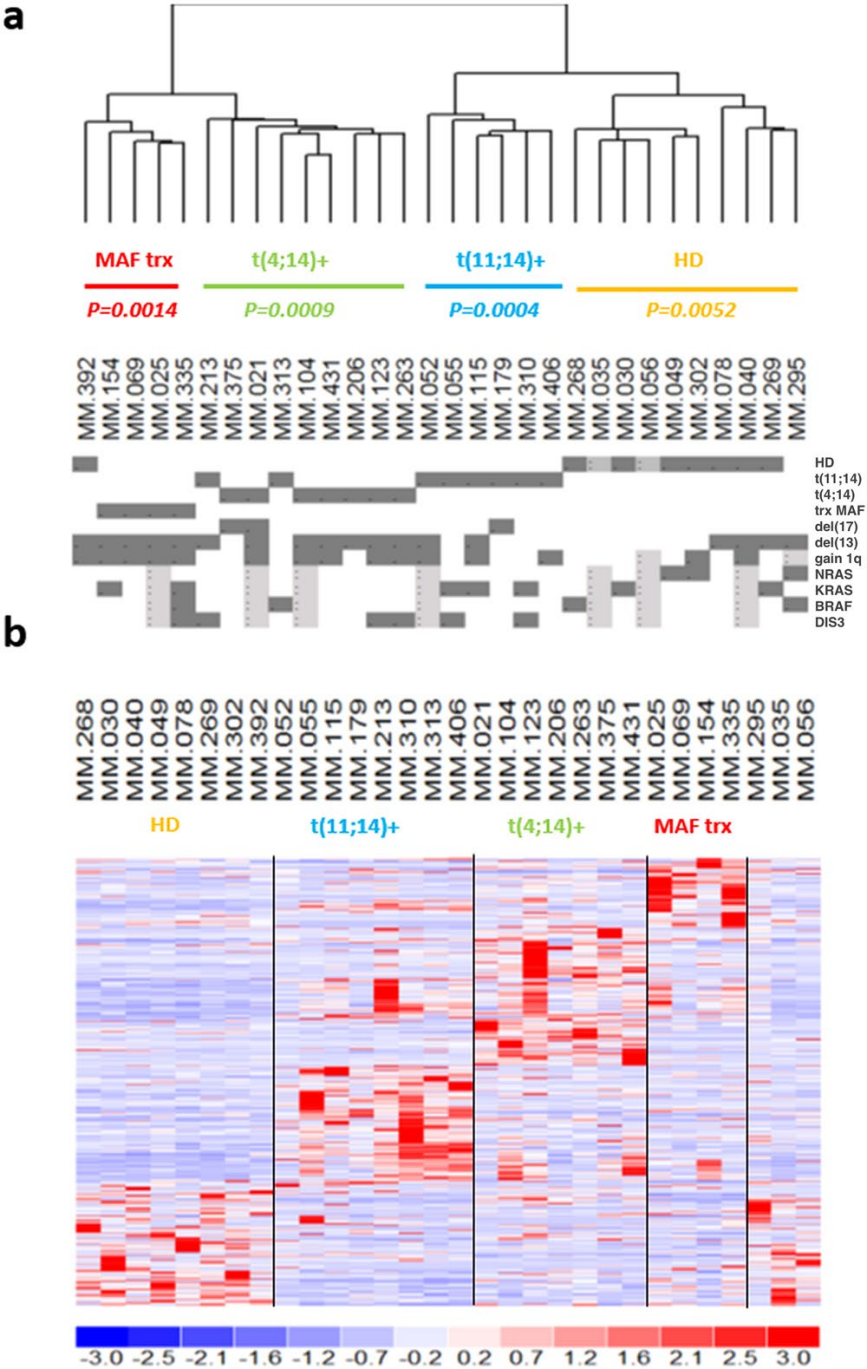
LncRNAs expression profiling in multiple myeloma. (**a**) Hierarchical agglomerative clustering of the 30 patients based on the 500 most variably expressed lncRNAs. Under each branch, hyperdiploid status (HD), t(11;14), t(4;14) or MAF translocation (trx) occurrence are specified with the corresponding *p*-values. For each MM sample, positivity (grey), negativity (white) or “not determined” (light grey) for the different molecular features are indicated. (**b**) Heatmap of the most differentially expressed lncRNAs (rows) in the 30 MM patients (columns) stratified into five groups according to their molecular features. The color scale spans the relative transcript expression changes standardized on the variance.

**Figure 2:**
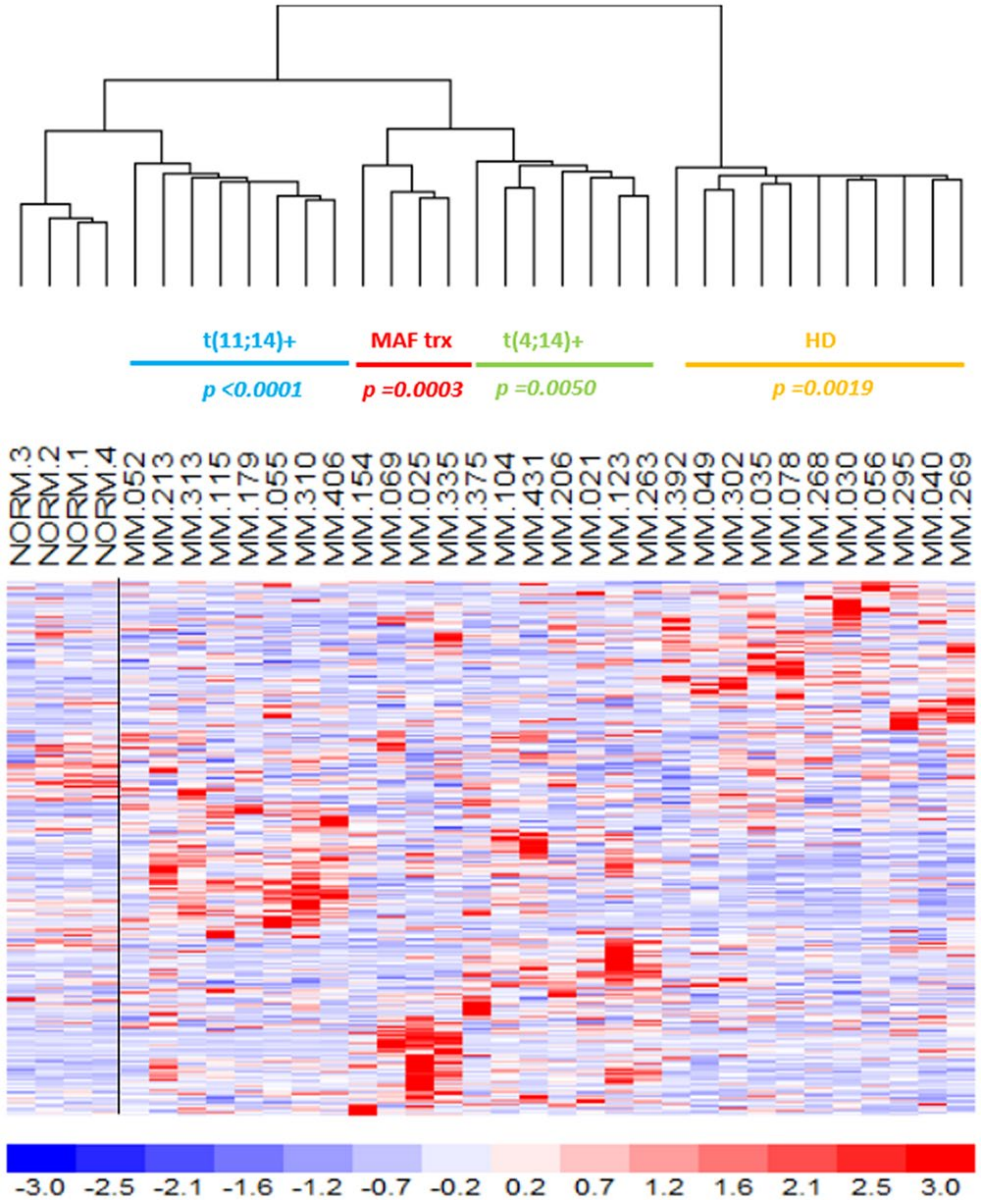
LncRNAs expression validation by microarray analysis. Hierarchical clustering (Pearson’s correlation and centroid as distance and linkage methods) of the 30 samples and 4 normal controls profiled on GeneChip® Human Gene 2.0ST arrays according to the expression values of the 262-lncRNA list. Under each branch, hyperdiploid status (HD), t(11;14), t(4;14) or MAF translocation (MAF trx) are specified with the corresponding *p*-values.

To identify MM patient subgroups, we used an unsupervised-learning method based on expression data. This analysis showed clusters of common global lncRNAs transcriptional patterns that were associated with the major and prognostically relevant molecular features, namely t(11;14), t(4;14), *MAF* gene translocations or HD status. In fact, unsupervised analysis of the 500 lncRNAs with the highest variation coefficient clearly showed that MM molecular subtypes were mainly and significantly clustered together (Fig. 1a). Next, we compared the lncRNAs expression profiles of each subgroups against all the other samples. We found the significant deregulation of 150 lncRNAs (116 down- and 34 up-regulated) in MM samples with HD status; 118 lncRNAs (68 down- and 50 up-regulated) characterized patients with t(11;14) translocation; and 96 lncRNAs (34 downregulated and 62 upregulated) MM carrying t(4;14). Finally, 42 lncRNAs (26 downregulated and 16 upregulated) defined MM with translocated *MAF* gene. Overall, we identified 391 unique lncRNAs differentially expressed among the four MM subgroups (Fig. 1b and Supplementary Table S2). Because the 30 MM investigated by RNA-seq had been previously profiled onto GeneChip® Human Gene 2.0 ST array together with 4 normal control, we verified whether that 391-lncRNA signature could be validated in the same cohort of patients assessed with a different technique. To this end, we evaluated the expression of the 262 of 391 lncRNAs detectable by the arrays, equally annotated on unambiguous entries in GENCODE encyclopedia. Overall, the dendrogram generated on the 262-lncRNA list clearly distinguished the diverse molecular subtypes and the normal samples (Mantel-Haenszel chi-squared test *p* < 0.00001; Fig. 2).

**Figure 3:**
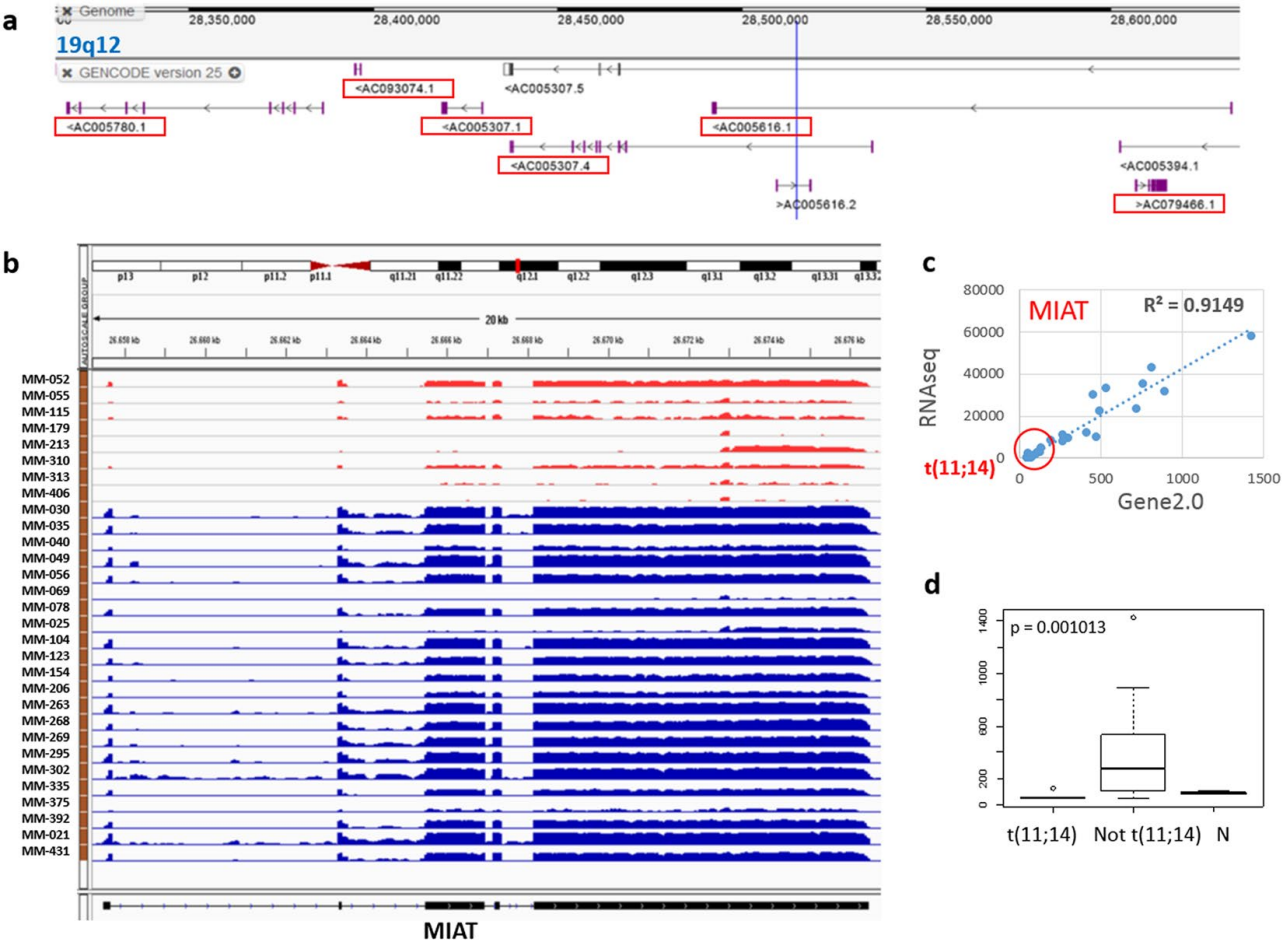
MM patients with t(11;14) downregulated a cluster of 6 transcripts at 19q12 and MIAT. (**a**) Screenshot of the 19q12 region from GENCODE browser of GRCh38/hg38 genome release. Red boxes indicated the lncRNAs significantly downregulated in t(11;14) MM. (**b**) Visualization of RNA-seq data: zoomed view of the *MIAT* lncRNA region; the coverage bigWig files generated using bamCoverage function in deeptools (http://deeptools.readthedocs.io/en/latest/content/tools/bamCoverage.html) and the human genome annotation file (GENCODE v.25) were loaded into the Integrated Genome Viewer (IGV [http://www.broadinstitute.org/igv/]. The y axis shows the scaled number of reads mapping to each location of the genome in the *MIAT* region (x axis); each lane represents a MM patient: samples t(11;14)-positive are shown in red. In order to compare samples, coverage values from all patients were group-scaled. (**c**) Correlation plot of *MIAT* expression in the 30 MM investigated by RNA-seq and GeneChip® Human Gene 2.0ST array. Red circle indicates t(11;14)-positive MM samples. (**d**) Box plot representation of *MIAT* expression in 8 t(11;14)-positive, 22 t(11;14)-negative MM patients and 4 normal controls (N) assessed by GeneChip® Human Gene 2.0ST array. P-value obtained by Kruskal-Wallis test.

The five most significant differentially expressed lncRNAs in each comparison are reported in Table 2. As regards MM carrying t(11;14), of note, the three most significantly downregulated lncRNAs belonged to a cluster of 6 transcripts located in a region of about 332 kb at 19q12 (Fig. 3a and Supplementary Table S2). In addition, this group showed also the downregulation of *MIAT* (Fig. 3b–d

**Table 2:** Top five lncRNAs significantly deregulated in distinct MM subgroups (Base Mean = median expression among samples; Stat = DEseq algorithm statistic).

	Ensembl gene id	Base Mean	Stat	Chr	Start position	End position	Gene name
**HD vs not HD**	ENSG00000271856	324.13	−6.23	3	108125821	108138610	LINC01215
	ENSG00000245330	13.68	−5.77	8	119867419	119874488	KB-1471A8.1
	ENSG00000235919	131.25	−5.52	1	155562042	155563944	ASH1L-AS1
	ENSG00000253364	22406.66	5.56	14	105644496	105649057	RP11-731F5.2
	ENSG00000260401	30.18	5.52	11	73238975	73242335	RP11-800A3.4
**t(11;14) vs not (11;14)**	ENSG00000267243 ENSG00000260725 ENSG00000260725	146 59.64 17.67	−10.06 −9.12 −7.19	19	28437060 28418483 28491864	28535277 28429490 28632728	AC005307.4 AC005307.1 AC005616.1
	ENSG00000260807	212.12	9.2	16	975761	981596	RP11-161M6.2
	ENSG00000174171	138.72	7.045	15	41892793	41898575	RP11-23P13.6
	ENSG00000260244	108.55	−6.82	4	155734448	155737062	AC005616.1
	ENSG00000225783	11802.7	−6.41	22	26646428	26676475	MIAT
**t(4;14) vs not (4;14)**	ENSG00000236154	6.21	10.18	10	69575807	69577154	RP11-343J3.2
	ENSG00000265778	2.72	8.08	18	76491652	76493918	RP11-17M16.2
	ENSG00000235597	4.64	7.84	2	104433267	104520832	LINC01102
	ENSG00000274307	3.05	7.13	15	25708470	25710869	RP11-345J18.2
	ENSG00000204832	58.68	−6.88	10	17386936	17413503	ST8SIA6-AS1
**trx MAF vs not trx MAF**	ENSG00000258776	30.07	11.51	14	56817570	56893710	RP11-1085N6.5
	ENSG00000234184	1252.1	−9.2	1	80535755	80646788	RP5-887A10.1
	ENSG00000261997	16.59	9.21	16	55538200	55542027	RP11-212I21.4
	ENSG00000270069	752.24	−7.28	X	45745211	45770274	MIR222HG
	ENSG00000185433	49.38	6.07	21	25385820	25431701	LINC00158

), a well-known lncRNA already reported as involved in different cancers.

### Identification of lncRNA signatures associated with genetic lesions or somatic mutations

Other genetic alterations occur at high frequency in MM and were associated by others and us to specific transcriptional profiles. Information was available for the 30 MM sample on numerical alterations and secondary events, i.e. somatic mutations (Table 1): therefore, we queried the RNA-seq dataset to evaluate the occurrence of differentially expressed lncRNAs in those MM genetic subtypes (Supplementary Table S3, with the exclusion of FAM46C and P53 due to the low number of samples).

In MM patients with 1q gain, we found the significant modulation of 12 lncRNAs (4 down- and 8- upregulated), two of which located on chromosome 1q. A list of 109 lncRNAs (31 down- and 78 up-regulated) distinguished del(13)-positive from wild-type patients; notably, 7 of 31 downregulated lncRNAs (23%) are located on chromosome 13. Finally, only two lncRNAs have been found downregulated in del(17)-MM.

As regards patients harboring the mutations of *BRAF*, *NRAS*, *KRAS*, or *DIS3* mutations, we identified the upregulation of 97 lncRNAs in DIS3 mutated samples, whereas 6 lncRNAs are upregulated in the samples grouped according to the presence of MAPK-pathway genes mutations.

### Selection of lncRNAs potentially relevant in MM

After the annotation process, we established a set of criteria to recognize the lncRNAs potentially relevant in MM biology. In particular, we investigated the levels of expression of lncRNAs localized in proximity to genes associated with MM, based on the recurrent evidence that the transcription of mRNAs and lncRNAs appears to be closely regulated, leading to a cis-regulatory relationship between the two transcripts^28–30^. For this purpose, a list of 707 genes mapped to GRCh38 primary assembly and associated with MM (from now on, referred as “MM-genes”) was downloaded from NCBI database (https://www.ncbi.nlm.nih.gov/gene, Supplementary Table S4). We analyzed the genomic context of the 707 MM-genes. In the boundaries of 409 of them, we found at least one of the 9,540 lncRNAs mapped within 4 Mb (Supplementary Table S4). Next, for each lncRNA/MM-gene couples we assessed the correlation of their expression levels and identified 43 significantly, and all positively, correlated pairs that involve 39 different genes and 35 different lncRNAs (combinations selected under criteria of Pearson coefficient >0.4 or <−0.4 and *p*-value < 0.01). Notably, all the 35 lncRNAs belong to the list of 391 unique lncRNAs differentially expressed among the four MM subgroups (Table 3

**Table 3:** Significant correlation among MM-genes and lncRNAs nearer than 4 Mb each other.

MM-Gene Symbol	description	Chr.	lncRNA	R	p-value	lncRNA in MM subgroup	
FBXW7	F-box and WD repeat domain containing 7	4q31	RP11-588K22.2	0.42	0.016	DW	t(11;14)
CCND2	cyclin D2	12p13	CCND2-AS1	0.95	1.50E-16	DW	
CUL4A	cullin 4A	13q34	GAS6-AS2	0.45	0.011	DW	
CCR2	C-C motif chemokine receptor 2	3p21	RP4-555D20.2	0.63	1.22E-04	DW	
WWOX	WW domain containing oxidoreductase	16q23	RP11-679B19.1 RP11-70D24.2	0.53 0.68	0.002 1.77E-05	DW DW	
MAF	MAF bZIP transcription factor	16q23	RP11-679B19.1 RP11-70D24.2	0.42 0.63	0.016 1.27E-04	DW DW	
RELN	reelin	7q22	CTB-107G13.1	0.42	0.017	UP	
PIK3CG	phosphatidylinositol-4,5-bisphosphate 3-kinase catalytic subunit gamma	7q22	CTB-107G13.1	0.45	0.009	UP	
PAX5	paired box 5	9p13	EBLN3	0.59	4.27E-04	UP	
KREMEN2	kringle containing transmembrane protein 2	16p13	AC005606.14	0.53	0.002	UP	
CCL2	C-C motif chemokine ligand 2	17q12	RP11-848P1.5	0.46	0.008	UP	
STAT3	signal transducer and activator of transcription 3	17q21	RARA-AS1	0.52	0.002	UP	
RARA	retinoic acid receptor alpha	17q21	RARA-AS1	0.70	8.49E-06	UP	
PMAIP1	phorbol-12-myristate-13-acetate-induced protein 1	18q21	RP11-299P2.2	0.42	0.017	UP	
HGF	hepatocyte growth factor	7q21	AC006145.4	0.45	0.009	DW	t(4;14)
AKT1	AKT serine/threonine kinase 1	14q32	RP11-731F5.2	0.43	0.013	DW	
DNMT1	DNA methyltransferase 1	19p13	CTD-3214H19.6	0.41	0.021	DW	
CD81	CD81 molecule	11p15	RP11-326C3.2	0.70	9.41E-06	DW	
PTGS2	prostaglandin-endoperoxide synthase 2	1q31	GS1-115G20.1	0.71	5.05E-06	UP	
TNFRSF1A	TNF receptor superfamily member 1A	12p13	RP5-1063M23.2	0.80	4.32E-08	UP	
PAK2	p21 (RAC1) activated kinase 2	3q29	LMLN-AS1	0.49	0.005	UP	
ADAM9	ADAM metallopeptidase domain 9	8p11	RP11-350N15.6	0.44	0.013	UP	
DICER1	dicer 1, ribonuclease III	14q32	RP11-433J8.1	0.41	0.018	UP	
PTPN6	protein tyrosine phosphatase, non-receptor type 6	12p13	RP5-1063M23.2	0.94	2.00E-15	UP	
BCL9	B-cell CLL/lymphoma 9	1q21	RP11-196G18.22	0.67	2.58E-05	DW	HD
HIST2H3C	histone cluster 2 H3 family member c	1q21	RP11-196G18.22	0.53	0.002	DW	
ILF2	interleukin enhancer binding factor 2	1q21	ASH1L-AS1	0.59	3.52E-04	DW	
DEPTOR	DEP domain containing MTOR interacting protein	8q24	KB-1471A8.1	0.73	2.38E-06	DW	
P2RX7	purinergic receptor P2X 7	12q24	RP11-347I19.7	0.49	0.005	DW	
CIITA	class II major histocompatibility complex transactivator	16p13	RP11-490O6.2	0.61	2.10E-04	DW	
NES	nestin	1q23	RP11-404F10.2	0.41	0.020	DW	MAF trx
FCRL4	Fc receptor like 4	1q23	RP11-404F10.2	0.43	0.015	DW	
DKK1	dickkopf WNT signaling pathway inhibitor 1	10q21	PRKG1-AS1	0.51	0.003	DW	
MBL2	mannose binding lectin 2	10q21	PRKG1-AS1	0.71	5.70E-06	DW	
NCAM1	neural cell adhesion molecule 1	11q23	RP11-629G13.1	0.53	0.002	DW	
XPO1	exportin 1	2p15	RP11-373L24.1 RP11-568N6.1	0.46 0.53	0.009 0.00	DW DW	
IFNL1	interferon lambda 1	19q13	PCAT19	0.40	0.023	DW	
TIMP1	TIMP metallopeptidase inhibitor 1	Xp11	RP6-99M1.3 MIR222HG	0.50 0.46	0.003 0.009	DW DW	
HAVCR2	hepatitis A virus cellular receptor 2	5q33	AC008697.1	0.47	0.007	UP	

The last column specifies for each lncRNA upregulation (UP) or downregulation (DW) in the specified MM group by DE-seq analysis.

).

## Discussion

In the present study, we have provided an unprecedented view of the lncRNAs expression in MM. As it occurred for mRNAs, miRNAs, and snoRNAs^6,7,31,32^, the natural clustering of whole lncRNAs transcriptional configuration is significantly associated with the major molecular prognostic alterations in MM, namely 11q13, 4p16, 16q23/20q12 chromosomal translocations, or HD status. In details, for each MM subtype we defined a specific and exhaustive lncRNAs expression signature based on the 14,202 lncRNAs currently annotated on GENCODE database. In a previous study concerning about 1,800 lncRNAs detectable by microarrays, we had reported a number of differentially expressed lncRNAs among the same MM subgroups. However, with very few exceptions (*MATN1-AS1* upregulated in MM with t(11;14), and *CRYM-AS1* and *LINC00158* upregulated in MAF translocated patients), RNA-seq data scarcely overlapped with our previous data. This discrepancy can be explained in all likelihood by two reason: first, the array annotation was based on a previous version of the LNCipedia repository (https://lncipedia.org)^33^ that had included pseudogenes and miscellaneous RNA within lncRNA transcripts, which are conversely excluded in the current study focused on transcripts annotated as “pure” long non-coding RNA. Second, very little is still known about the processing and the prevalence of alternative transcripts for many lncRNAs, whose “splicing” products are often roughly defined and/or based on predictions. While RNA-seq allowed to evaluate the non-coding genes in their full extension (according to the provided annotations), microarrays evaluation is probe-position dependent and might be therefore affected by the number of transcripts in the queried region. This last aspect undoubtedly reinforce the highest accuracy of RNA-seq data for complex transcriptome processing. We are aware that the number of samples analyzed in this study does not allow drawing definitive conclusion, all the more true in that myeloma patients may share different primary/secondary molecular alterations. We kept this in close consideration when the cohort was selected, aimed at being representative of the major genetic lesions and avoiding as much as possible that confounding variables might affect data in differential analysis (graphical legend to the Fig. 1a). To further overcome these limitations, lncRNAs expression evaluated by RNA-seq technology was validated by high-density arrays, overall leading to the definition of a comprehensive background for future investigations of lncRNAs in plasma cell dyscrasias.

Considering the lncRNAs expression signatures, no information is currently available for the majority of the lncRNAs identified. Among the five most significant lncRNAs found differentially expressed in each comparison (Table 2), the well-known lncRNA *MIAT* resulted specifically downregulated in MM carrying t(11;14). Originally identified within a susceptible locus for myocardial infarction on chromosome 22q12.1, *MIAT* was then characterized as the RNA component of specific nuclear bodies where it may affect RNA splicing, ultimately regulating gene expression^34^. Recently Sattari *et al*.^35^ found *MIAT* upregulation in leukemia/lymphoma lymphoid lineage with mature B-cell phenotype; interestingly, they demonstrated a higher incidence of *MIAT* upregulation in aggressive types of CLL and worst clinical outcome. In addition, this study described a positive feedback regulatory loop between *MIAT* and *OCT4*, acting on evading apoptotic cell death in malignant mature B cells. Overall, these findings suggest an involvement of *MIAT* in supporting proliferation of the malignant mature B-cells. In this perspective, lower *MIAT* expression in t(11;14)-positive patients might be associated with the better prognosis associated with this MM subtype^36^.

Among the most significant lncRNAs defining the signature of HD-MM, we found the downregulation of *ASHL1-AS1* and *KB-1471A8.1*. Both lncRNAs resulted also from the analyses aimed at identifying lncRNAs that are located in proximity to, and concordantly expressed with genes important in the context of MM pathology (Table 3). In details, *ASHL1-AS1* maps 1,89 Mb telomeric to *ILF2*, overexpressed in MM as a result of 1q21 amplification. *ILF2* overexpression deregulates homologous recombination (HR) by stabilizing the mRNA splicing of critical HR effectors, which enables genomic instability, promotes adaptive mechanisms to genotoxic stress, and enhances cell survival, thereby promoting drug resistance and disease progression^37^. As regards *KB-1471A8.1*, it maps at 8q24 antisense to the 5′ region of *DEPTOR*, a crucial gene in the maintenance of the terminal differentiation of MM cells^38^. Since the overexpression of *DEPTOR* in MM has been associated with *MAF* translocations and the expression of *CCND1* and *CCND3* genes^39^, the downregulation of *KB-1471A8.1* in HD-MM further suggest a cis-regulatory connection with *DEPTOR*.

Finally, among the most significant lncRNAs deregulated in *MAF* translocated MM, our data unraveled the *MIR222HG* sequence, from the maturation of which originate microRNAs 221 and 222 that were found accordingly downregulated in this MM subtype^31^. *MIR222HG* is located at Xp11 about 1.7 Mb telomeric to the *TIMP1* gene encoding an inhibitor of metalloproteinases. As the balance between metalloproteinases and their inhibitors, including TIMP1, largely influences cell adhesion, proteolytic shedding, and cell signaling, it will be of great interest to clarify the putative regulation of *TIMP1* expression by *MIR222HG*.

Overall, to our knowledge our study provides the first comprehensive catalogue by RNA-seq of lncRNAs in MM, which is highly beneficial as a valuable reference for future research  on their involvement into the pathogenesis of the disease.

## Methods

### Samples

The molecular features of the 30 patients at diagnosis included in the study cohort are shown in Table 1. PCs purification has been previously described and led to >90% enrichment in all samples [Mattioli, Oncogene 2005]. According to already reported FISH procedure^40^, eight samples showed the t(11;14) translocation, with the consequent overexpression of either CCND1, and a non-hyperdiploid (HD) status; 8 MM were HD; seven patients showed high CCND2 levels and the presence of the t(4;14) translocation; and four expressed the highest levels of CCND2 in association with either the t(14;16) or t(14;20) translocations. Information on 17p13 and 13q14 deletions, and gain of 1q arm was also available. Mutation of *BRAF*, *NRAS*, *KRAS*, *P53*, *FAM46C* and *DIS3* were investigated by next-generation sequencing^41–44^. Written informed consent was obtained from all patients in accordance with the declaration of Helsinki. The study was approved by the Ethical Committee of the University of Milan (N°24/15, May 06 2015).

### RNA sequencing

Total RNA was extracted from purified PCs by using Trizol reagent. Quantitative assessment of the RNA was performed using Nanodrop ND-1000 Biophotometer (NanoDrop Technologies): the minimum OD 260/280 ratio to be considered acceptable is 1.98–2.10. Four-hundred ng of total RNA were used to prepare paired-end (PE) cDNA libraries using the TruSeq® RNA Sample Preparation kit for total RNA (Illumina). The libraries were sequenced to obtain strand-specific 100 bp PE reads on a HiSeq. 2000 (Illumina). Reads were aligned to the human genome using STAR under default conditions and Gencode v25 GTF file. STAR aligner was based on splice junctions from the Ensembl database version 87. Transcript abundance was estimated using featureCounts (default parameters). FPKM (Fragments Per Kilobase Million) quantification was performed on sorted BAM files using *cufflinks* default procedure. Differentially expressed genes were identified using DeSeq at FDR < 0.01, provided that expression across the whole dataset was not null. Quality Control (QC) analysis was performed using *multiqc* tool and the QC metrics were comparable for all samples. The annotation allowed to detect 14,202 lncRNAs, including the following Gencode biotypes: lincRNA, antisense, bidirectional promoter lncRNA, sense intronic, sense overlapping, 3′ overlapping ncRNA. The expression filter retained 9,540 lncRNAs in our dataset.

### Gene Expression Profiling

Thirty MM samples and four normal controls (purchased from Voden, Medical Instruments IT) were profiled onto GeneChip® Human Gene 2.0 ST arrays (Affymetrix Inc., Santa Clara, CA). Total RNA samples were processed according to manufacture’s procedure. Normalized expression values were obtained using Robust Multi Array Average (RMA) procedure. A custom annotation pipeline was applied that combined GENCODE v25 (Ensembl v87) annotations with the CDF (Chip Definition File) version 21 for gene annotations freely available at http://brainarray.mbni.med.umich.edu/Brainarray/Database/CustomCDF/21.0.0/genecodeg.asp, in order to withdraw probes that map to regions where ambiguous detection due to transcript overlap might occur. Therefrom, the expression levels of Ensembl genes specific for 10138 unique lncRNAs were obtained.

All the data have been deposited in the NCBI Gene Expression Omnibus database (GEO; http://www.ncbi.nlm.nih.gov/geo) and are accessible under accession #GSE109116.

### Statistical analysis

Pearson’s correlation as distance and centroid linkage were used in hierarchical agglomerative clustering analysis. Conventional statistical tests were applied as reported in the manuscript using standard packages for R software.

## Electronic supplementary material

Supplementary Tables
