# Supplementary material for: A compendium of long non-coding RNAs transcriptional fingerprint in multiple myeloma
Source: Sci Rep. 2018 Apr 26;8:6557. doi: 10.1038/s41598-018-24701-8 (PMC5920050; doi:10.1038/s41598-018-24701-8)
Supplement: Supplementary file 1 — Supplementary Tables [file 41598_2018_24701_MOESM1_ESM.pdf]

SUPPLEMENTARY TABLES

A compendium of long non-coding RNAs transcriptional fingerprint in multiple myeloma

Domenica Ronchetti[1,2], Luca Agnelli[1,2], Alessandro Pietrelli[3,4], Katia Todoerti[1], Martina Manzoni[1,2], Elisa Taiana[1,2], Antonino Neri[1,2*]

[1]Department of Oncology and Hemato-oncology, University of Milan, Italy
[2]Hematology, Fondazione Cà Granda IRCCS Policlinico, Milan, Italy
[3]Internal Medicine and Metabolic Diseases, Fondazione IRCCS Ca' Granda Ospedale Policlinico, Milan, Italy
[4]Bioinformatic Unit, Istituto Nazionale Genetica Molecolare, Milan, Italy

Domenica Ronchetti and Luca Agnelli contributed equally to this work

Corresponding author:
Antonino Neri, MD, PhD
Dept. of Oncology and Hemato-oncology
Università degli Studi di Milano
via Festa del Perdono 7, 20122 Milano, Italy
Phone: +39 02-50320420
Fax: +39 02-50320403
e-mail: antonino.neri@unimi.it

Supplementary Table S1. Highly expressed lncRNAs in MM (average read counts >5000)

Gene ID	gene name	chromosome	Average read counts >5000	Normalized counts
ENSG00000253364	RP11-731F5.2	14q32	223595	176406.6
ENSG00000245532	NEAT1	11q13	87317	84136.2
ENSG00000251562	MALAT1	11q13	75663	74937.8
ENSG00000253701	AL928768.3	14q32	20424	17091.8
ENSG00000260032	NORAD	20q11	13435	13109.6
ENSG00000225783	MIAT	22q12	12577	11802.7
ENSG00000203709	C1orf132	1q32	8779	8373.1
ENSG00000226777	KIAA0125	14q32	7824	6305.8
ENSG00000253352	TUG1	22q12	6739	6285.7
ENSG00000229807	XIST	Xq13	6042	6118.2
ENSG00000281649	EBLN3	9p13	5417	5182.3
ENSG00000270164	LINC01480	19q13	5060	4711.7

Supplementary Table S2. LncRNAs differentially expressed in MM in association with HD, t(11;14), t(4;14), or MAF translocation (Base Mean=median expression among samples; Stat= DEseq algorithm statistic).

HD vs notHD q-value <0.01						
**lncRNA**	**base Mean**	**log2 Fold Change**	**stat**	**chr.**	**start position**	**end position**
LINC01215	324.134	-2.817	-6.228	3	108125821	108138610
KB-1471A8.1	13.684	-3.490	-5.774	8	119867419	119874488
ASH1L-AS1	131.253	-1.836	-5.523	1	155562042	155563944
RP11-731F5.2	22406.661	4.189	5.561	14	105644496	105649057
RP11-800A3.4	30.180	2.787	5.517	11	73238975	73242335
RP11-196G18.22	373.508	-1.527	-5.393	1	149844498	149849024
RP11-191G24.1	24.333	-2.777	-5.251	1	51518309	51561629
RP11-424M21.1	66.331	4.033	5.257	4	151799500	151801348
AC007879.5	23.030	-2.317	-4.997	2	207239864	207529795
SGMS1-AS1	295.838	-1.422	-4.980	10	50624951	50641451
ZBTB40-IT1	53.918	2.052	5.018	1	22517474	22519708
RP11-428G5.5	37.948	-3.618	-5.041	12	31877079	31887203
RP11-192H23.6	17.666	-1.854	-5.055	17	28607963	28609730
RP11-196G18.24	67.600	-1.337	-4.978	1	149845816	149846486
RP11-452L6.7	26.221	-2.024	-4.926	16	31508471	31509256
CTD-3126B10.1	6.876	-3.202	-4.921	16	2597881	2599718
LOXL1-AS1	13.080	-3.706	-4.832	15	73908071	73928248
RP11-490O6.2	22.349	-1.908	-4.818	16	11741910	11744506
CTD-2017D11.1	77.348	-2.431	-4.846	19	24033445	24066909
RP11-464F9.20	28.186	-2.043	-4.820	10	73625996	73626790
RP11-11N7.4	16.855	-2.847	-4.838	1	244864738	244865272
RP11-347I19.7	40.166	-1.931	-4.819	12	121800797	121803403
HIPK1-AS1	31.861	-2.588	-4.746	1	113924000	113929492
RP11-160H22.5	13.597	-2.623	-4.667	1	174115300	174160004
RP5-827C21.4	113.152	-1.563	-4.626	1	234372807	234373593
RP11-9M16.2	14.397	-1.779	-4.630	9	114396724	114398503
RP11-282I1.1	10.907	2.229	4.618	10	123356450	123517708
CTD-2036P10.5	5.361	1.914	4.604	15	42724102	42724922
MCF2L-AS1	17.758	-3.428	-4.596	13	112967484	112968824
MATN1-AS1	322.372	-1.573	-4.480	1	30718504	30726827
RP11-371A19.2	7.092	-3.255	-4.506	10	23343957	23345181
PARD3-AS1	7.650	-3.091	-4.523	10	34815767	34816386
EPHA1-AS1	15.167	-3.472	-4.493	7	143407813	143523449
AC093673.5	43.628	-2.174	-4.535	7	143379692	143380495
RP11-21L19.1	5.833	-3.529	-4.484	11	14262846	14273691
CTD-2288F12.1	11.272	-1.682	-4.501	16	18926863	18937043
HMGN3-AS1	25.709	-1.685	-4.503	6	79233718	79236797
RP11-51J9.5	52.146	-1.925	-4.511	8	30155830	30156232
AC011330.13	6.540	1.818	4.482	15	43642389	43643023
CTB-58E17.1	142.904	-1.093	-4.517	17	38702452	38704747
CTD-2267D19.3	37.778	-2.555	-4.472	17	40360655	40364693

CTD-3154N5.2	13.586	1.498	4.458	15	74598919	74599397
CTC-546K23.1	48.513	3.579	4.430	5	121199704	121357398
AC142472.6	50.840	-1.626	-4.433	17	45146730	45148470
SPATA3-AS1	2.948	3.717	4.417	2	230984368	230996032
AATBC	2.722	2.544	4.412	21	43805758	43812567
RP11-613M10.6	79.770	1.888	4.382	9	37509150	37510299
RP5-965G21.4	40.272	-1.802	-4.384	20	25239007	25245229
RP4-559A3.6	8.754	-3.346	-4.352	1	225936411	225937557
RP11-385D13.3	57.775	-1.813	-4.350	17	15530773	15531089
RP11-46J23.1	23.366	-2.227	-4.356	4	70703747	70704491
RP11-94B19.6	3.748	3.554	4.334	18	76173304	76177738
RP11-197N18.7	32.357	-2.945	-4.319	12	123081384	123084744
RP11-156E8.1	61.294	-1.639	-4.318	1	244969350	244971088
IQCH-AS1	567.656	1.378	4.309	15	67403619	67521844
CTD-3075F15.1	21.066	-1.546	-4.284	14	75176929	75177418
RP11-298J20.4	151.631	-0.898	-4.265	10	124917143	124942881
RP11-252A24.3	12.581	-2.283	-4.246	16	74367462	74369826
AC004076.5	27.395	1.270	4.249	19	57449689	57453011
RP11-425M5.7	21.067	-1.551	-4.242	20	37526642	37527060
RP11-670E13.6	20.233	-2.418	-4.235	17	56914186	56914533
AC062017.1	32.911	-1.742	-4.212	2	239401436	239402364
AC009120.4	5.126	-2.583	-4.189	16	74054151	74296762
NAALADL2-AS2	66.633	3.801	4.180	3	175234861	175271096
DNAJC9-AS1	14.077	-1.234	-4.180	10	73247360	73276984
RP11-46H11.3	3.724	-3.467	-4.152	12	132911470	132914732
RP11-848P1.2	74.280	-1.637	-4.145	17	30971652	30973312
RP11-646I6.5	23.664	-1.408	-4.132	4	56387625	56388153
RP11-329N22.1	8.937	-2.825	-4.111	1	38474875	38496034
RP11-468H14.2	4.089	-3.361	-4.114	8	17131181	17149789
CTD-2313J17.5	43.599	1.890	4.088	15	92779757	92781492
RP1-40E16.12	20.648	-1.810	-4.076	6	3068045	3068894
RP11-298E9.7	17.126	-3.182	-4.072	10	3134566	3145166
RP11-686D22.4	5.562	1.496	4.062	17	35403837	35404373
RP13-516M14.1	210.536	-1.651	-4.055	17	82293716	82294910
LINC00869	278.349	-1.153	-4.050	1	149606334	149679523
SMAD5-AS1	4.110	1.779	4.036	5	136129507	136134890
RP5-864K19.4	20.070	-2.102	-4.034	1	38860000	38919396
RP11-481J2.3	14.223	-1.680	-4.029	16	58392153	58392807
RP11-574F21.2	19.982	-1.928	-4.019	1	160261744	160262778
RP13-1032I1.7	120.354	-1.117	-4.018	17	81701324	81703300
RP11-540B6.6	433.988	0.790	4.013	15	30926514	30928407
RP11-46A10.5	73.026	-0.946	-4.006	1	180944042	180976482
RP11-339B21.10	22.178	1.317	3.994	9	128431598	128432006
RP11-488L18.8	64.586	-1.089	-3.983	1	247210691	247241823
RP11-370I10.12	28.013	-2.055	-3.982	12	48198387	48202031
LINC01422	124.568	-1.327	-3.968	22	26858634	26865786
RP11-74C1.4	12.748	-1.411	-3.968	1	151540516	151561855
RP11-760H22.2	155.039	-2.030	-3.972	8	120052180	120056201

RP11-297C4.1	2.458	3.337	3.970	16	30498766	30499554
RP5-933K21.3	3.544	-2.444	-3.975	6	157323964	157324477
RP11-483I13.5	31.000	-1.543	-3.953	1	108199926	108201491
RP11-109G23.3	10.435	-2.543	-3.940	4	78773654	78775973
RP4-545K15.5	154.669	-1.368	-3.935	X	101627868	101628523
ALMS1-IT1	126.578	-2.140	-3.932	2	73456764	73459484
RP11-705C15.3	133.223	-1.542	-3.922	12	9658567	9662085
RP11-48B3.5	93.804	-1.395	-3.910	8	80484561	80485619
SAPCD1-AS1	82.723	-1.509	-3.906	6	31764310	31765588
RP11-428J1.4	24.836	1.407	3.904	6	5031756	5054423
CTD-2095E4.5	35.211	-2.148	-3.899	17	32127595	32128454
RP11-1006G14.1	7.686	1.524	3.893	15	72615810	72618250
LINC01625	2.862	2.982	3.885	6	139468995	139474596
RP11-93O14.2	12.099	-1.757	-3.885	16	46660696	46661591
RP11-77E14.2	6.784	1.569	3.879	9	7786105	7786688
RP1-265C24.8	7.112	-2.230	-3.864	6	28136849	28139678
AC079630.4	137.553	-2.285	-3.846	12	40186009	40224915
CTD-2193G5.1	2.246	3.132	3.842	5	103528434	103541985
RP11-1E4.1	4.292	-2.984	-3.838	8	10050485	10054254
RP5-858B6.3	2.581	-2.855	-3.829	1	230874846	230879015
NDUFV2-AS1	47.909	-1.221	-3.823	18	9121265	9136645
AC004540.5	22.418	-1.823	-3.816	7	26398593	26494256
AC069513.4	5.008	-2.276	-3.796	3	195655565	195657927
RP11-37B2.1	336.764	-1.067	-3.799	8	89609409	89757727
RP11-803P9.1	21.619	1.677	3.795	3	197789700	197790369
AC090952.5	3.779	-2.593	-3.787	3	14648194	14649432
ZNF674-AS1	236.024	-1.062	-3.776	X	46545493	46548408
RP5-882C2.2	68.226	-1.207	-3.773	17	44221401	44223710
RP11-126O1.6	80.589	-1.583	-3.767	18	58659858	58660524
RP11-220I1.5	4.139	-2.588	-3.762	9	37078813	37079776
RP13-942N8.1	43.639	-1.022	-3.754	12	123363868	123366113
RP11-190A12.8	21.210	-2.584	-3.749	1	159866954	159867685
CTC-429P9.3	235.587	0.913	3.737	19	16633797	16635269
ZMIZ1-AS1	7.316	-2.594	-3.728	10	78943328	79067895
ERICD	41.229	-1.907	-3.722	8	140636281	140638283
RP1-317E23.3	25.646	-1.373	-3.718	1	25816749	25820797
RP11-122K13.12	140.118	-1.140	-3.714	10	133295187	133295977
RAP2C-AS1	93.668	-1.009	-3.700	X	132217147	132432862
AC007879.2	11.218	-2.069	-3.704	2	207186717	207236066
RP11-197K3.1	9.040	-2.747	-3.704	3	114445521	114529452
RP11-307C19.1	2.246	1.839	3.702	15	77525540	77534110
RP11-455F5.5	281.802	-1.170	-3.698	16	30183505	30184957
RP11-259K5.2	58.778	-0.961	-3.702	3	37241789	37244177
RP4-647J21.1	41.730	-2.408	-3.695	7	44958999	44960909
RP11-73E17.2	189.213	-1.664	-3.693	14	34874343	34876459
SERPINB9P1	70.414	-2.024	-3.689	6	2854657	2881407
TAT-AS1	11.582	-1.488	-3.685	16	71565789	71578187
AC144449.1	9.557	1.267	3.680	2	149587196	149848233

RP11-473I1.9	5230.145	-1.425	-3.679	16	9104848	9113181
RP11-462B18.2	4.900	1.502	3.672	9	32840598	32841762
RP11-554J4.1	74.123	-1.146	-3.671	2	55617909	55618373
LPP-AS2	8.919	-1.271	-3.667	3	188151206	188154057
RP11-131L12.2	9.649	-1.631	-3.663	12	118375350	118376275
RP13-516M14.10	15.043	-2.532	-3.663	17	82244770	82245591
RP11-629O1.2	26.083	-2.014	-3.649	8	133573183	133573861
CTD-2036P10.6	4.602	1.451	3.651	15	42726583	42727211
RP11-80I3.1	218.485	2.668	3.648	9	26066675	26118408
RP11-536C5.7	44.871	-2.049	-3.644	1	160202199	160208869
RP5-855D21.3	38.526	-1.594	-3.642	8	233119	233692
RP11-44F14.5	7.580	-1.993	-3.637	16	53448748	53449590
RP11-399O19.9	7.057	-2.770	-3.635	10	89015836	89017059

**t(11;14) vs not(11;14) q-value <0.01**						
**lncRNA**	**base Mean**	**log2Fold Change**	**stat**	**chr.**	**start position**	**end position**
AC005307.4	145.996	-7.566	-10.057	19	28437060	28535277
RP11-161M6.2	212.117	6.949	9.205	16	975761	981596
AC005307.1	59.645	-7.196	-9.119	19	28418483	28429490
AC005616.1	17.670	-5.551	-7.194	19	28491864	28632728
RP11-23P13.6	138.721	5.059	7.045	15	41892793	41898575
RP11-588K22.2	108.557	-5.195	-6.824	4	155734448	155737062
MIAT	11802.673	-3.984	-6.415	22	26646428	26676475
RP11-424M21.1	104.804	-5.431	-6.350	4	151799500	151801348
RP11-428G5.5	35.845	3.721	5.818	12	31877079	31887203
CTD-2537O9.1	7.898	-5.010	-5.754	11	84720826	84800701
SOS1-IT1	72.248	-1.929	-5.576	2	38992279	38993857
GAS6-AS2	52.112	-2.748	-5.545	13	113864168	113866833
EBLN3	5182.307	1.007	5.549	9	37079857	37090507
RP11-572O17.1	29.396	1.311	5.334	4	1712821	1713622
MATN1-AS1	310.713	1.664	5.313	1	30718504	30726827
RP11-314B1.2	23.254	-3.996	-5.293	2	225698514	225703654
AC144831.1	23.501	3.135	5.061	17	83104255	83106910
CTC-523E23.11	22.592	-3.538	-5.051	19	34849278	34860576
MIR503HG	28.900	-3.904	-5.028	X	134543337	134546711
AC022182.1	41.458	2.453	5.026	8	60910053	60966557
PIK3IP1-AS1	8.732	2.710	4.964	22	31292499	31338021
AC092635.1	18.873	4.604	4.980	2	13537673	13609168
AC005519.4	72.400	1.345	4.964	14	74289127	74294425
AC092198.1	6.140	4.234	4.924	X	40009276	40012182
AC007966.1	11.083	-2.342	-4.925	2	185719874	185740479
OVAAL	22.396	-4.360	-4.902	1	180558976	180566518
CTD-2341M24.1	38.662	1.804	4.906	14	85934710	86129778
RP11-128A17.1	8.452	-4.465	-4.890	15	37099339	37100173
RP11-568N6.1	618.322	-2.295	-4.880	2	64522187	64524093
RP11-679B19.1	11.801	-4.317	-4.848	16	79202624	79206739
DGUOK-AS1	14.585	-3.121	-4.831	2	73947642	73981441

RP11-73G16.2	34.947	-4.034	-4.819	4	151919468	151943933
CCND2-AS1	5.173	-4.369	-4.823	12	4248765	4276184
SPATA41	8.985	-3.312	-4.805	15	100344457	100349655
AC016730.1	33.351	4.122	4.734	2	13723048	13758152
RP11-268G12.3	21.743	-3.861	-4.734	X	27042907	27176298
AC079466.1	30.816	-4.201	-4.740	19	28606688	28615229
RP4-555D20.4	29.932	-3.016	-4.744	3	43998081	43999149
AC159540.1	181.013	-1.554	-4.712	2	97416165	97433527
HHIP-AS1	14.618	2.497	4.694	4	144642922	144661357
AC005780.1	3.807	-4.247	-4.684	19	28316705	28386204
RP11-734K23.9	23.959	-1.643	-4.679	2	97281904	97291780
RP11-598F7.3	603.419	-3.134	-4.638	12	106524	111850
AC139100.4	23.289	-2.125	-4.627	18	80161752	80162413
RP11-70D24.2	5.581	-4.249	-4.631	16	79605802	79606605
AC084809.2	31.359	1.606	4.562	17	32876759	32878637
RP11-8P13.5	3.712	-3.796	-4.544	12	32820142	32820567
RP11-108P20.4	3.932	2.873	4.530	18	58813880	58834364
RP11-96B5.3	11.555	-3.708	-4.515	10	51062579	51068553
RP11-114H23.1	2.300	4.104	4.498	12	75563202	75984015
RP11-73O6.3	38.214	-3.364	-4.449	6	130133410	130146179
RP11-114H23.2	2.042	4.011	4.426	12	75694010	75698816
LINC01229	6.330	-3.733	-4.412	16	79676108	79807922
RP11-268G12.1	19.624	-3.646	-4.405	X	27174920	27398997
ELFN1-AS1	27.907	-3.274	-4.397	7	1738630	1742291
CTD-2377D24.6	16.353	-3.919	-4.381	17	48646923	48707346
RP11-425D10.10	9.250	1.233	4.340	6	109382795	109383666
CTC-523E23.1	9.256	-3.338	-4.302	19	34811589	34814345
AC005394.1	3.203	-3.958	-4.283	19	28602379	28648303
RP11-147L13.8	20.868	2.138	4.257	17	68189884	68192802
CTB-107G13.1	10.756	3.977	4.253	7	103445207	103514007
LINC01234	77.846	-2.601	-4.246	12	113679459	113773683
KCNQ5-IT1	11.553	2.087	4.240	6	72630495	72678558
RP11-473M20.16	6.524	2.053	4.233	16	3156736	3157483
RP11-848P1.5	5.517	2.124	4.213	17	31090787	31095450
TRIM31-AS1	2.753	-3.889	-4.208	6	30105240	30114724
AC108004.3	42.519	-1.661	-4.199	17	404468	414023
KB-1460A1.5	116.810	-1.903	-4.194	8	101166805	101169629
CTD-2201G3.1	8.400	-2.479	-4.181	5	113323028	113437174
RP4-742J24.2	6.657	-3.335	-4.178	20	11909404	11918677
LINC00882	11.957	-2.289	-4.141	3	106836811	107240641
LINC01481	153.096	1.521	4.142	12	69904033	70243360
CTD-2015H6.3	33.547	-2.697	-4.128	5	80482293	80488063
RP11-3D4.4	3.808	-2.873	-4.084	15	33850538	33851178
SCARNA10	5.960	2.178	4.066	12	6510275	6510522
RPPH1	35.496	2.133	4.044	14	20343048	20343685
AC116614.1	25.647	-3.841	-4.006	2	949627	950274
CTB-58E17.1	143.553	0.942	4.006	17	38702452	38704747
SLC16A1-AS1	180.911	0.934	3.997	1	112956415	113047055

AC061961.2	2.117	3.408	3.983	2	154696462	154697817
MIR3681HG	2.156	2.455	3.973	2	11848622	12578348
USP2-AS1	10.421	-2.734	-3.962	11	119381778	119526664
CTD-2002H8.2	47.855	1.277	3.949	14	58264662	58269681
AC010761.8	12.457	1.605	3.947	17	28721487	28722877
KB-1958F4.1	4.205	2.427	3.941	8	97144170	97144723
CTD-2583P5.3	14.588	3.562	3.937	16	12759282	12761162
DKFZp779M0652	3.774	-2.644	-3.934	11	45771432	45772358
RARA-AS1	15.682	1.647	3.921	17	40340867	40343136
MIR600HG	1023.915	1.327	3.918	9	123109494	123115477
RAMP2-AS1	25.562	-3.068	-3.897	17	42753914	42761257
SERPINB9P1	68.206	1.987	3.888	6	2854657	2881407
RP11-90D4.3	3.924	-2.723	-3.869	12	8321876	8329614
RP4-555D20.2	1405.279	-2.951	-3.865	3	44117299	44122365
RP11-299P2.2	30.485	2.631	3.851	18	63207622	63208168
LINC00957	96.693	-1.815	-3.842	7	44039171	44042306
LINC01515	10.544	-2.768	-3.832	10	65570338	65768835
FLJ27354	98.457	1.192	3.831	1	89583241	89632894
EDNRB-AS1	31.585	-2.044	-3.818	13	77828222	77833950
TFAP2A-AS1	40.020	-2.938	-3.815	6	10409340	10416446
RP11-386I14.4	152.700	2.199	3.814	1	78004346	78004554
SMPD5	14.750	-2.780	-3.797	8	144049129	144051522
AC007128.1	7.839	-1.956	-3.795	7	8262264	8344516
LARS2-AS1	2.688	2.481	3.790	3	45483974	45509545
AC007009.1	7.065	-2.027	-3.789	7	8262233	8262821
GDNF-AS1	10.573	-3.442	-3.784	5	37811589	37953827
RP1-45N11.1	2.880	-3.066	-3.784	6	90345219	90362632
RP11-654D12.2	1.912	2.581	3.776	12	89882665	89989289
AC005606.14	2.640	2.159	3.774	16	1984877	1990357
BOLA3-AS1	62.294	-1.713	-3.772	2	74148009	74150061
CTD-2516F10.2	485.507	1.229	3.767	11	7427266	7512515
RP11-5G9.5	5.274	1.632	3.768	13	43877715	43878163
RP11-278C7.4	18.121	0.940	3.763	12	32725248	32725660
AC079630.2	14.687	3.415	3.754	12	40156239	40167707
LINC01289	180.387	3.563	3.750	8	63769430	63785501
AC093074.1	2.015	-3.543	-3.752	19	28394851	28396428
CTD-2310F14.1	7.296	-1.775	-3.751	5	56927874	56929573
RP11-9N20.3	3.604	-3.184	-3.748	3	123715851	123716399
RP11-295H24.5	2.653	-2.957	-3.695	15	49353485	49354034

**t(4;14) vs not(4;14) q-value <0.01**						
**lncRNA**	**base Mean**	**log2Fold Change**	**stat**	**chr.**	**start position**	**end position**
RP11-343J3.2	6.206	6.721	10.181	10	69575807	69577154
RP11-17M16.2	2.722	4.639	8.083	18	76491652	76493918
LINC01102	4.639	6.580	7.838	2	104433267	104520832
RP11-345J18.2	3.052	4.548	7.134	15	25708470	25710869
ST8SIA6-AS1	58.676	-6.057	-6.879	10	17386936	17413503

FAM201A	10.163	4.744	6.744	9	38620474	38624990
RP5-942I16.1	2.396	5.222	6.572	7	69595958	69597448
RP11-713P17.3	1.748	5.339	6.037	11	134032272	134046849
RP11-461O7.1	1.266	4.787	6.040	16	56092987	56191094
RP11-109M17.2	32.729	-5.310	-6.057	9	10948372	10948481
AP001065.15	5.221	4.545	6.009	21	44485577	44490288
AC108142.1	2.235	5.338	5.748	4	181874438	182145249
RP11-744N12.3	16.636	-4.295	-5.729	11	128629653	128686922
RP11-439C15.4	2.196	4.853	5.708	8	1972570	1974637
AC005301.9	86.897	4.313	5.642	22	16601911	16615111
XXYLT1-AS2	14.302	-5.110	-5.624	3	195147871	195152790
DPP10-AS1	1.493	4.994	5.416	2	115130935	115161343
AC007392.3	0.931	4.379	5.369	2	66574030	66730157
RP11-648K4.2	22.813	-4.500	-5.361	15	87432058	87703852
LINC01013	16.836	-4.913	-5.310	6	132131915	132169374
FAM95C	21.781	3.084	5.278	9	38540567	38545372
XXbac-BPG308K3.5	1.117	3.951	5.251	6	28837869	28839006
RP11-527D7.1	0.912	4.650	5.180	1	241424292	241433492
RP11-14N7.2	1.406	5.131	5.182	1	144227030	144250288
C20orf197	8.091	2.232	5.110	20	60055925	60072953
RP5-1063M23.2	1.292	4.526	5.092	12	3318718	3325343
AF127936.3	208.146	-2.983	-5.016	21	14746762	14753863
DLX6-AS1	4.194	4.079	5.009	7	96955141	97014065
LMLN-AS1	2.797	3.650	4.958	3	198038321	198039234
CTA-384D8.35	9.404	-2.779	-4.923	22	50542305	50542906
CTA-280A3.2	35.333	-4.342	-4.892	22	47916728	47926264
LINC00939	0.954	4.773	4.841	12	125958688	125983374
AC006145.4	18.380	-4.280	-4.804	7	82009177	82029955
RP11-96D1.11	9.752	-4.079	-4.763	16	68225969	68229145
LL22NC03-N14H11.1	12.204	-4.141	-4.758	22	15823197	15823890
RP11-326C3.2	166.140	-3.516	-4.738	11	287305	288987
CTC-526N19.1	3.011	3.960	4.733	19	33906352	33908391
RP4-639F20.1	23.898	-4.024	-4.698	1	94927566	94963270
HOXA-AS2	5.850	2.196	4.690	7	27107777	27134302
RP11-548B3.3	4.419	2.950	4.681	9	69428248	69428721
RP11-350N15.6	1.607	3.850	4.662	8	38408048	38408742
CTD-2154I11.2	8.543	-2.329	-4.557	5	95701249	95732295
HAGLROS	2.926	3.586	4.531	2	176177717	176179008
AC226118.1	2.871	3.943	4.525	7	379359	382712
RP11-356N1.2	6.107	2.697	4.469	1	108040263	108076020
GS1-115G20.1	8.705	3.209	4.480	1	184408337	184412360
HIPK1-AS1	30.097	2.431	4.470	1	113924000	113929492
TSPEAR-AS1	9.118	3.946	4.475	21	44506807	44516575
RP11-713P17.4	0.626	4.392	4.445	11	134035832	134037107
RP11-92G12.3	4.929	2.059	4.420	1	200669507	200694250
TARID	1.357	3.641	4.374	6	133502252	133892802
NR2F2-AS1	3.529	3.774	4.368	15	96110040	96327361
RP11-23P13.6	40.920	-3.523	-4.350	15	41892793	41898575

RP11-439C15.5	0.549	4.257	4.326	8	1974818	1975314
CTD-2081C10.1	9.729	-3.238	-4.282	5	53776644	53819686
RP11-568J23.8	10.786	-3.483	-4.241	16	85697335	85697868
RP11-272L14.2	2.618	3.554	4.201	13	106506046	106506713
RP11-93B14.9	160.224	-2.166	-4.204	20	62648961	62650767
AC005220.3	25.332	-2.755	-4.190	20	53940160	53942508
RP11-13E5.2	12.174	-3.424	-4.187	X	126109762	126115562
AC079466.1	30.816	-3.911	-4.190	19	28606688	28615229
PCAT6	73.517	1.474	4.172	1	202810954	202812156
RP3-512B11.3	1.193	4.194	4.167	6	7540451	7541338
RP11-81H3.2	28.740	-3.632	-4.144	12	74133166	74402535
RP4-790G17.7	7.487	3.414	4.146	1	150255095	150257286
CCDC183-AS1	153.956	-1.389	-4.132	9	136803927	136808848
AC008440.10	6.828	3.907	4.134	19	53864763	53866140
RP11-80I3.1	247.077	-3.346	-4.126	9	26066675	26118408
AC093901.1	5.890	1.828	4.093	2	118132128	118186386
CELSR3-AS1	2.857	-3.450	-4.088	3	48663776	48669174
RP11-731F5.2	21305.120	-3.624	-4.088	14	105644496	105649057
RP11-433J8.1	3.931	3.144	4.093	14	96592733	96595762
RP11-793J2.1	11.050	2.449	4.080	18	63966406	63970181
RP11-317N12.1	34.345	-3.323	-4.071	8	33604856	34039008
RP11-304L19.12	6.536	2.116	4.073	16	2235689	2236913
AC079776.1	1.510	3.221	4.042	2	129869193	129877553
AC073133.1	2.449	3.373	4.036	7	156437789	156445588
KB-68A7.1	27.726	1.995	4.009	21	44477850	44478493
RP5-1011O1.2	9.070	-3.511	-3.989	1	192517639	192567217
AC009312.1	1.567	3.386	3.989	2	117757563	117804174
CTD-3214H19.6	10.592	-2.078	-3.987	19	7633766	7636990
LOXL1-AS1	12.385	3.072	3.982	15	73908071	73928248
CYP4A22-AS1	1.438	2.708	3.972	1	47096653	47179271
RP11-35O15.1	1.531	2.430	3.969	15	98646951	98647371
LINC01152	0.600	4.056	3.948	17	72030291	72041297
RP11-94I2.4	24.327	1.108	3.935	1	148021850	148025931
DKFZp779M0652	3.774	1.968	3.929	11	45771432	45772358
RP11-469N6.1	7.122	-3.669	-3.926	11	134735596	134763810
LINC01608	5.484	-3.816	-3.910	8	110937690	111027433
RP11-399K21.14	2.694	3.038	3.910	10	75409157	75411842
RP11-518L10.5	87.180	0.678	3.865	1	244375100	244409592
CNTN4-AS1	17.358	-2.750	-3.859	3	3039033	3069242
CTD-2631K10.1	3.033	1.905	3.842	5	72794405	72816565
RP11-350N15.3	1.394	3.982	3.840	8	38400536	38401683
RP11-775D22.2	5.676	-3.121	-3.833	7	130141731	130142615
RP11-385F5.4	55.188	0.997	3.833	1	236540094	236550280

**trx MAF vs not trx MAF q-value <0.01**						
**lncRNA**	**base Mean**	**log2Fold Change**	**stat**	**chr.**	**start position**	**end position**
RP11-1085N6.5	30.072	9.569	11.509	14	56817570	56893710

RP5-887A10.1	1252.096	-7.708	-9.197	1	80535755	80646788
RP11-212I21.4	16.594	4.099	9.205	16	55538200	55542027
MIR222HG	752.242	-6.340	-7.279	X	45745211	45770274
LINC00158	49.383	5.973	6.072	21	25385820	25431701
AC008697.1	38.091	5.495	5.940	5	159310933	159805536
SLC25A25-AS1	751.629	-1.693	-5.713	9	128108581	128118693
GAS6-AS1	196.701	-4.358	-5.348	13	113815630	113845744
LINC00930	13.528	4.075	5.311	15	92567818	92572042
CRYM-AS1	7.387	3.623	5.212	16	21300849	21318591
KIAA0125	6305.787	-5.747	-5.181	14	105917979	105932642
PCAT19	23.086	-5.189	-5.138	19	41454169	41500649
CACNA1C-AS2	39.840	-3.749	-4.998	12	2668500	2672220
RP6-99M1.3	70.081	-5.166	-4.981	X	45764772	45765299
RP11-404F10.2	314.675	-2.573	-4.667	1	160670778	160699761
CACNA1C-AS1	62.907	-3.732	-4.637	12	2676001	2691200
LINC00877	132.039	-2.571	-4.513	3	72035300	72279503
RP11-1084E5.1	50.083	-3.516	-4.531	8	108895029	109063417
RP11-568N6.1	618.322	2.518	4.515	2	64522187	64524093
LACTB2-AS1	42.727	-1.752	-4.502	8	70608577	70663279
PTOV1-AS1	74.039	1.977	4.475	19	49838639	49851676
RP11-70D24.3	7.449	5.058	4.452	16	79603572	79604177
CTA-384D8.34	36.988	3.487	4.440	22	50542650	50543011
MIR600HG	1023.915	-2.022	-4.400	9	123109494	123115477
LINC01140	61.760	-2.011	-4.389	1	87129765	87169198
KIAA1614-AS1	11.952	2.223	4.357	1	180949699	180954887
RP11-1246C19.1	62.429	-1.672	-4.286	7	1464497	1467522
PRKG1-AS1	52.472	-3.154	-4.261	10	52230742	52314128
LINC01011	32.373	2.433	4.223	6	2987967	2991173
TMEM147-AS1	813.549	-1.334	-4.202	19	35540738	35546029
RP11-373L24.1	478.149	-3.497	-4.208	2	60925909	60931610
RP11-340F14.6	9.895	2.495	4.169	12	121190868	121191518
AC078842.3	12.226	-4.911	-4.147	7	137344930	137354483
AC012123.1	24.975	-4.954	-4.153	18	32769795	32774413
LINC01229	24.837	4.400	4.101	16	79676108	79807922
AC010149.4	9.263	-4.815	-4.061	2	230520594	230580006
RP1-240B8.3	16.446	-4.808	-4.013	6	61630233	61681049
RHOXF1-AS1	18.217	-3.901	-3.974	X	120036236	120146854
RP11-629G13.1	14.292	-4.751	-3.959	11	112959279	112963460
AC079150.3	39.545	3.593	3.924	2	153337705	153357422
RP11-404O13.5	52.307	-4.024	-3.914	1	158197922	158203877
RP11-456K23.1	125.289	1.915	3.865	18	44676927	44679717

Supplementary Table S3. LncRNAs differentially expressed in MM in association with 1q gain, del(13), del(17), DIS3 or KRAS mutation (Base Mean=median expression among samples; Stat= DEseq algorithm statistic).

**1q-gain vs not 1q-gain q-value <0.01**						
**lncRNA**	**base Mean**	**log2Fold Change**	**stat**	**chr.**	**start position**	**end position**
RP11-799B12.4	11.641	2.336	5.023	18	24159509	24162211
RP11-299P2.2	29.818	-2.390	-5.096	18	63207622	63208168
LINC01268	32.259	-1.793	-4.356	6	113868013	113873351
AC013463.2	16.244	-1.647	-4.283	2	164912312	165208261
RP11-731F5.1	13.398	1.998	4.327	14	105601472	105605357
RHPN1-AS1	4.966	2.185	4.437	8	143366631	143368548
AC145124.2	4.833	1.806	4.358	8	12194467	12196280
RP5-1139B12.2	27.023	1.694	4.271	1	228270443	228274397
XXbac-B444P24.14	4.466	1.911	4.289	22	20320739	20321203
RP11-897M7.4	2.316	-2.116	-4.265	12	131447337	131455436
RP11-679B19.1	12.266	2.257	4.546	16	79202624	79206739
RP4-559A3.6	12.511	2.086	4.211	1	225936411	225937557

**del(13) vs not del(13) q-value <0.01**						
**lncRNA**	**base Mean**	**log2Fold Change**	**stat**	**chr.**	**start position**	**end position**
CCND2-AS1	5.173	3.525	6.636	12	4248765	4276184
LVCAT1	31.852	3.238	6.244	4	44016861	44022063
RP11-428K3.1	80.351	2.064	5.203	1	178724306	178726285
RP11-212P7.2	194.423	1.345	5.195	7	128524016	128531069
RP11-299P2.2	30.485	-2.507	-5.057	18	63207622	63208168
RP1-239B22.5	42.434	-2.301	-4.994	11	17380649	17383531
AC093627.9	11.333	2.625	4.849	7	135853	149466
RP11-658F2.8	484.153	-1.011	-4.926	11	66666036	66668374
RP11-318A15.2	3.941	2.402	4.862	17	76671942	76673658
RP11-792A8.4	85.236	1.013	4.839	7	66739829	66740385
CTD-2270P14.5	54.896	2.247	4.880	16	2777319	2780568
PARD6G-AS1	96.352	1.777	4.797	18	80147924	80178432
AC016700.5	11.692	1.780	4.754	2	70124036	70125317
AC145124.2	4.914	2.057	4.757	8	12194467	12196280
LINC01006	72.195	2.066	4.740	7	156472196	156640654
FLJ21408	9.423	2.421	4.727	16	27268205	27290492
LINC01569	80.555	1.102	4.715	16	4245825	4253789
CTD-2020K17.3	46.489	-1.170	-4.692	17	45238028	45241734
WAC-AS1	388.306	1.107	4.681	10	28522652	28532743
RP11-656D10.3	4.135	2.234	4.661	1	40493157	40508661
RP11-380B4.3	17.966	-1.537	-4.625	13	32782874	32788178
RP11-90M2.5	4.676	-1.807	-4.594	13	48041751	48042184
LOH12CR2	21.289	1.588	4.572	12	12355406	12357067
RP11-190A12.8	20.147	2.297	4.571	1	159866954	159867685
RP11-967K21.1	52.751	1.463	4.538	12	28163298	28190738
AC142528.1	7.835	1.762	4.498	2	3519275	3523197
XXbac-B444P24.14	5.188	2.202	4.498	22	20320739	20321203

EXOC3-AS1	50.069	1.390	4.473	5	441498	443160
AC139100.3	5.806	2.429	4.467	18	80183680	80202992
LINC00571	47.579	-1.258	-4.455	13	38050817	38143232
C1orf220	103.587	1.524	4.441	1	178542752	178548889
AC105760.2	15.036	1.863	4.416	2	237059434	237085817
RP11-809N8.4	9.483	-1.280	-4.406	11	73405297	73410682
TNK2-AS1	33.089	-1.559	-4.388	3	195908076	195911257
AP003900.6	24.843	2.387	4.382	21	10328411	10342737
RP11-944C7.1	5.964	-2.247	-4.374	14	89628921	89642671
AC009950.2	12.119	-2.184	-4.363	2	230121370	230174223
AC023590.1	8.605	-2.268	-4.309	8	118282139	118400605
RP11-517P14.2	40.564	1.026	4.316	10	43420738	43422100
AP003774.1	33.063	-1.511	-4.317	11	64325050	64329504
RP4-559A3.6	5.852	2.324	4.315	1	225936411	225937557
RP11-61J19.5	38.636	2.028	4.267	1	212557833	212559731
RP11-799B12.4	11.298	2.202	4.269	18	24159509	24162211
RP11-307C12.12	22.728	1.192	4.266	1	154961825	154962623
RP5-1061H20.4	7.281	2.128	4.249	1	229258281	229271028
RP11-248J18.3	20.850	-1.311	-4.233	14	50723777	50724272
RP11-563N4.1	11.750	1.655	4.214	2	32165046	32165757
LINC00482	4.808	2.017	4.187	17	81303771	81309248
RP11-412D9.4	24.473	1.649	4.171	12	106954029	106955497
JHDM1D-AS1	154.670	1.479	4.157	7	140177261	140179640
ANKRD10-IT1	279.624	-1.152	-4.097	13	110894639	110899172
FARP1-AS1	9.557	-1.701	-4.084	13	98435405	98435840
RP11-29H23.4	6.983	2.120	4.083	1	155609776	155610380
CCND2-AS2	1.419	2.196	4.083	12	4252735	4275065
RP11-672L10.6	12.704	1.793	4.086	18	813274	813756
RP11-379H18.1	260.985	1.172	4.078	7	35695214	35699413
CTD-2267D19.3	35.802	1.877	4.051	17	40360655	40364693
RP13-467H17.1	6.534	1.972	4.044	8	142403652	142407028
GAS6-AS1	196.701	-1.877	-4.038	13	113815630	113845744
RP11-25H12.1	11.266	2.166	4.028	4	65998846	66150012
ENTPD1-AS1	119.967	0.826	4.016	10	95753206	96090238
RP3-395M20.12	134.256	1.757	4.010	1	2546465	2547460
RP11-306I1.2	5.054	2.171	3.998	1	165598463	165623331
XXbac-BPG294E21.9	8.638	1.939	3.986	6	33437363	33454453
AC009133.17	144.052	1.639	3.981	16	29745247	29748299
RP3-518E13.2	11.258	-2.150	-3.977	1	175307218	175335459
RP11-370I10.12	27.492	1.625	3.974	12	48198387	48202031
AC013463.2	16.333	-1.567	-3.961	2	164912312	165208261
LIFR-AS1	4.321	2.150	3.955	5	38556786	38671216
SCARNA9	43.182	-1.356	-3.956	11	93721513	93721865
RP11-848P1.5	5.517	-1.709	-3.952	17	31090787	31095450
RP11-165E7.1	9.578	1.790	3.954	16	5010909	5043999
CEP83-AS1	15.942	1.602	3.950	12	94460003	94462484
RP11-407N17.5	52.577	1.245	3.940	14	39265703	39267061
OSER1-AS1	212.918	1.137	3.925	20	44210960	44226027
XX-C283C717.1	9.860	-1.329	-3.919	22	50314631	50316008
ABHD11-AS1	7.104	1.838	3.914	7	73735038	73736054

RP11-290D2.6	884.006	-1.177	-3.909	13	45340039	45341183
GS1-124K5.4	57.207	0.879	3.895	7	66493706	66495474
CTD-2516F10.2	485.507	-1.099	-3.882	11	7427266	7512515
CTD-3065J16.9	18.332	1.281	3.884	8	144078002	144079265
CTD-2006C1.12	29.707	-0.901	-3.885	19	11939959	11953381
CTD-2033C11.1	9.094	1.964	3.881	5	65924629	65925135
RP11-408A13.4	34.683	-1.082	-3.889	9	14588797	14590065
RP11-567M16.6	69.752	1.304	3.894	18	79677287	79679358
LINC00235	40.836	1.607	3.866	16	525155	527407
CTD-2619J13.17	33.601	1.702	3.854	19	58428632	58431148
LINC01202	3.193	2.158	3.844	5	161910252	162001196
RP13-977J11.2	7.139	1.939	3.832	12	132186735	132189695
RP11-333E1.1	26.912	1.101	3.790	17	5192084	5248069
AC124944.3	5.908	-1.539	-3.780	3	195912049	195913986
RP11-1008C21.2	11.715	1.986	3.783	15	38069481	38072959
RP5-921G16.2	14.465	-1.569	-3.777	7	124337380	124349860
PTOV1-AS1	74.039	1.254	3.777	19	49838639	49851676
CTD-2256P15.2	19.703	1.348	3.771	5	10352701	10353601
LINC01144	43.945	1.325	3.773	1	45303910	45305619
RP11-191L17.1	8.348	1.697	3.767	2	48440043	48440597
CTC-575N7.1	5.044	-2.045	-3.760	5	129500361	129905917
RP11-708J19.1	68.465	1.597	3.759	3	47379089	47380999
RP1-45C12.1	21.274	1.953	3.731	1	171247580	171251794
LINC01018	7.916	2.000	3.732	5	6582136	6588499
RP11-107N15.1	4.692	1.980	3.732	2	202032770	202033537
RP5-1065J22.8	35.341	1.298	3.738	1	109087971	109090858
RP11-54C4.3	40.195	1.098	3.737	5	151770242	151771508
RP5-1024G6.5	11.235	1.332	3.726	1	53238610	53242783
RP11-467L13.7	96.486	1.398	3.721	12	31729117	31731204
YTHDF3-AS1	26.687	1.147	3.715	8	63167725	63168442
CTC-304I17.6	5.496	-2.082	-3.711	17	33827728	34079100
EBLN3	5182.307	-0.720	-3.702	9	37079857	37090507

del(17) vs not del(17) q-value <0.01

lncRNA	base Mean	log2Fold Change	stat	chr.	start position	end position
FIRRE	104.10595	-3.4114042	-5.3090374	X	131691530	131830643
RP11-266K4.9	42.152941	-2.9294602	-4.5715825	12	8235415	8242564

DIS3 mut vs DIS3 WT q-value <0.01

lncRNA	base Mean	log2Fold Change	stat	chr.	start position	end position
RP11-341N2.1	24.656	2.831	6.371	2	242087351	242088457
FLJ21408	7.943	2.877	6.051	16	27268205	27290492
RP11-147L13.2	30.349	2.280	5.962	17	68246629	68247938
RP11-197N18.7	27.684	2.828	5.892	12	123081384	123084744
STAM-AS1	19.402	2.615	5.761	10	17641284	17643878
RP11-377G16.2	14.646	2.992	5.658	4	80183280	80190169
RP11-158I9.8	60.516	2.077	5.555	11	118791254	118793137
RP11-708J19.1	69.107	2.253	5.485	3	47379089	47380999

RP11-707G18.1	70.586	2.381	5.415	12	25385670	25386241
RP11-11M20.4	22.557	1.932	5.352	20	62066830	62068437
CTD-2371O3.3	117.071	2.108	5.300	11	9459556	9460702
RP11-191L17.1	9.739	2.250	5.269	2	48440043	48440597
CTD-2515O10.5	16.332	2.287	5.272	16	30064306	30064825
RP11-65N13.8	6.092	2.730	5.234	9	125241663	125257018
CTD-2270P14.5	52.931	2.398	5.149	16	2777319	2780568
RP11-214N9.1	11.917	1.914	5.117	2	9555899	9556775
CAPN10-AS1	110.313	1.473	5.102	2	240582700	240586699
RP11-467P9.1	15.829	2.355	5.023	2	71067519	71068125
RP11-107N15.1	21.457	2.634	4.963	2	202032770	202033537
LA16c-358B7.4	7.632	2.302	4.948	16	1305547	1309413
CTD-2619J13.23	5.800	2.303	4.906	19	58475355	58475763
CTD-3193O13.12	18.034	2.266	4.857	19	7898804	7903542
CTD-2095E4.5	32.423	2.071	4.827	17	32127595	32128454
CTA-204B4.2	62.086	1.523	4.793	8	140505813	140508043
RP11-81A1.6	276.115	1.007	4.739	15	79920195	79922455
CTD-2023M8.1	11.672	1.855	4.614	5	18704433	18746173
CTD-2547G23.4	63.208	1.795	4.615	16	25257952	25261066
RP4-635E18.7	42.168	2.052	4.591	1	11068471	11073097
CTD-2302E22.4	5.663	2.405	4.563	14	63543569	63544664
RP11-533E19.7	55.928	1.675	4.504	1	179881607	179882595
RP11-554J4.1	76.489	1.294	4.517	2	55617909	55618373
ZNF674-AS1	251.118	1.149	4.506	X	46545493	46548408
RP11-383C5.5	16.185	1.936	4.492	10	125718771	125719365
CTA-292E10.6	134.264	1.563	4.469	22	28800683	28848559
AC007950.2	9.028	1.611	4.417	15	63500737	63503083
IFT74-AS1	4.846	2.085	4.399	9	26955780	26956295
CTD-2619J13.27	4.508	2.325	4.398	19	58500505	58501137
AP001437.1	14.632	1.788	4.372	21	37365477	37365932
HIPK1-AS1	62.429	2.228	4.361	1	113924000	113929492
CTD-2526A2.2	6.385	2.315	4.357	17	80453735	80454729
TTLL7-IT1	142.599	2.013	4.343	1	83979118	83984300
RP11-188D8.1	12.807	1.748	4.333	1	117364899	117365473
RP11-629O1.2	27.160	1.953	4.269	8	133573183	133573861
RP5-881P19.7	21.839	2.057	4.255	1	228073909	228076550
RP11-692D12.1	13.963	1.551	4.250	4	73259209	73317953
ATXN2-AS	3.520	1.588	4.259	12	111599498	111600256
RP11-334J6.7	6.442	2.135	4.244	9	131497479	131500191
RP6-109B7.5	2.872	1.930	4.236	22	46053093	46053560
RP11-61J19.5	33.161	2.013	4.219	1	212557833	212559731
RP11-108M9.6	5.311	2.079	4.208	1	16904339	16904776
KMT2E-AS1	247.797	1.407	4.215	7	105013425	105014321
CTB-55O6.10	9.412	2.019	4.208	19	14119106	14119537
MIR4453	470.999	1.522	4.195	4	152536264	152539263
AC009133.15	5.229	2.134	4.189	16	29806496	29807732
RP11-102M11.2	6.758	2.014	4.158	3	136752630	136755780
CTD-2033C11.1	8.914	2.107	4.156	5	65924629	65925135
XXbac-B444P24.14	4.791	2.037	4.155	22	20320739	20321203
MGC45922	13.872	1.516	4.146	19	50808874	50818880

RP11-306I1.2	5.202	2.180	4.127	1	165598463	165623331
RP11-705O1.8	12.102	1.781	4.127	18	268148	270278
RP11-159D12.10	3.061	2.121	4.071	17	58076891	58083204
RP11-603J24.14	7.579	1.768	4.037	12	56150796	56158220
RP11-474G23.3	8.812	1.691	4.018	2	68252870	68253848
LINC00235	38.208	1.764	3.996	16	525155	527407
CTD-2562J15.6	19.385	1.937	3.995	19	16283359	16324514
FTX	225.271	0.954	3.988	X	73963955	74293574
RNF139-AS1	159.388	0.855	3.978	8	124462485	124474576
ZNF667-AS1	24.364	2.109	3.968	19	56477250	56500666
RP11-318E3.9	39.336	1.321	3.963	10	5813985	5814441
RP11-46F15.2	6.226	1.779	3.934	1	3623190	3624743
RP11-336K24.12	31.408	1.647	3.929	1	155978799	155982986
RP11-303E16.7	2.743	2.023	3.916	16	81077319	81078861
RP11-195C7.1	11.547	1.215	3.901	1	176207648	176229330
RP11-285G1.14	6.169	1.907	3.900	10	44958917	44959458
AP001619.2	13.854	1.425	3.883	21	41874756	41877613
AC073934.6	6.033	1.805	3.864	7	127644685	127652012
LINC01089	551.111	1.180	3.871	12	121795267	121803906
RP11-61F12.1	6.606	1.840	3.876	16	84594393	84596826
CTD-3194G12.2	1.418	2.050	3.872	17	36722583	36726340
RP11-49I11.4	9.273	1.997	3.864	18	36189824	36190272
RP5-821D11.7	24.864	1.433	3.868	22	41831215	41834665
MIR378D2	2.595	1.963	3.859	8	93915734	93916682
RP11-297A16.2	4.476	1.721	3.839	10	35098006	35127020
RP11-154J22.1	30.620	1.493	3.839	15	48312353	48331856
CTD-3222D19.12	10.495	1.577	3.839	19	16542746	16544814
RP11-563N4.1	11.479	1.634	3.834	2	32165046	32165757
RP11-343H5.6	7.435	1.603	3.810	1	206634649	206635622
RP11-190C22.8	3.322	1.932	3.819	3	119497678	119498181
XXbac-BPG294E21.9	8.123	1.854	3.811	6	33437363	33454453
CTD-2650P22.1	3.909	1.958	3.817	15	52010999	52019095
RP11-96O20.5	10.281	1.706	3.815	15	45585757	45586304
RP11-55K13.1	4.601	1.864	3.811	16	81310731	81313423
RP5-855D21.3	37.445	1.458	3.790	8	233119	233692
CTD-2007H13.3	16.387	1.504	3.747	5	98929171	98995013
NFYC-AS1	60.547	1.169	3.734	1	40690380	40692066
SNHG19	204.765	1.682	3.736	16	2154797	2155358
ILF3-AS1	848.215	1.106	3.734	19	10651862	10653844

MAPK-pathway genes mut vs WT q-value <0.01						
**lncRNA**	**base Mean**	**log2Fold Change**	**stat**	**chr.**	**start position**	**end position**
RP11-486L19.2	29.783	2.873	5.115	16	84192558	84197053
RP11-367F23.1	57.400	3.052	5.314	9	90957260	90965393
RP11-375O18.2	20.060	1.908	4.630	9	79505804	79532342
RP11-486L19.2	29.783	2.818	4.918	16	84192558	84197053
RP11-734I18.1	43.469	2.470	4.876	4	31997397	32155406
RP11-1149M10.2	4.209	2.500	4.889	8	81154279	81164599

Supplementary Table S4. MM-genes list downloaded from NCBI database (https://www.ncbi.nlm.nih.gov/gene, November 2017)

Symbol	description	map location	start position	end position	lncrna ± 4 Mb
**AATF**	apoptosis antagonizing transcription factor	17q12	36948875	37056871	**CTB-58E17.1; RARA-AS1**
**ABCB1**	ATP binding cassette subfamily B member 1	7q21.12	87503863	87713323	
**ABCB4**	ATP binding cassette subfamily B member 4	7q21.12	87399461	87475864	
**ABCC1**	ATP binding cassette subfamily C member 1	16p13.11	15949577	16143074	**CTD-2583P5.3; CTD-2288F12.1**
**ABCG2**	ATP binding cassette subfamily G member 2 (Junior blood group)	4q22.1	88090264	88231417	
**ACKR1**	atypical chemokine receptor 1 (Duffy blood group)	1q23.2	159204013	159206500	**ASH1L-AS1; RP11-404O13.5; RP11-404F10.2**
**ACKR3**	atypical chemokine receptor 3	2q37.3	236537131	236582358	
**ACP5**	acid phosphatase 5, tartrate resistant	19p13.2	11574660	11579008	**CTD-3214H19.6**
**ADAM10**	ADAM metallopeptidase domain 10	15q21.3	58595204	58749978	
**ADAM9**	ADAM metallopeptidase domain 9	8p11.22	38996771	39105261	**RP11-350N15.3; RP11-350N15.6**
**ADAMTS13**	ADAM metallopeptidase with thrombospondin type 1 motif 13	9q34.2	133414339	133459403	**CCDC183-AS1**
**AGO2**	argonaute 2, RISC catalytic component	8q24.3	140520158	140642406	**SMPD5**
**AICDA**	activation induced cytidine deaminase	12p13.31	8602166	8612970	**SCARNA10; RP11-90D4.3**
**AKAP7**	A-kinase anchoring protein 7	6q23.2	131125698	131283535	**RP11-73O6.3; LINC01013; TARID**
**AKT1**	AKT serine/threonine kinase 1	14q32.33	104769349	104795743	**RP11-731F5.2; KIAA0125**
**ALB**	albumin	4q13.3	73404255	73421412	**RP11-46J23.1**
**ALDH1A1**	aldehyde dehydrogenase 1 family member A1	9q21.13	72900662	72953317	**BANCR; RP11-548B3.3**
**AMBP**	alpha-1-microglobulin/bikunin precursor	9q32	114060127	114078472	**RP11-9M16.2**
**ANG**	angiogenin	14q11.2	20684177	20694186	**RPPH1**
**ANGPT1**	angiopoietin 1	8q23.1	107249482	107498055	**RP11-1084E5.1; LINC01608**
**ANGPT2**	angiopoietin 2	8p23.1	6499651	6563420	
**ANKHD1**	ankyrin repeat and KH domain containing 1	5q31.3	140401814	140539856	
**ANKRD11**	ankyrin repeat domain 11	16q24.3	89267619	89490561	**RP11-568J23.8**
**ANXA2**	annexin A2	15q22.2	60347151	60398025	
**ANXA2R**	annexin A2 receptor	5p12	43039080	43040345	

**APEX1**	apurinic/apyrimidinic endodeoxyribonuclease 1	14q11.2	20455131	20457772	**RPPH1**
**APOC1**	apolipoprotein C1	19q13.32	44914247	44919349	**PCAT19**
**AREG**	amphiregulin	4q13.3	74445098	74455009	**RP11-46J23.1**
**ARMC9**	armadillo repeat containing 9	2q37.1	231198546	231394991	**AC010149.4**
**AURKA**	aurora kinase A	20q13.2	56369389	56392337	**AC005220.3; C20orf197**
**B2M**	beta-2-microglobulin	15q21.1	44711487	44718159	**RP11-23P13.6**
**BAG6**	BCL2 associated athanogene 6	6p21.33	31639028	31660900	**XXbac-BPG308K3.5; TRIM31-AS1**
**BCL2**	BCL2, apoptosis regulator	18q21.33	63123346	63319778	**RP11-299P2.2; RP11-793J2.1**
**BCL2L1**	BCL2 like 1	20q11.21	31664452	31723999	
**BCL2L11**	BCL2 like 11	2q13	111120914	111168445	**DPP10-AS1**
**BCL3**	B-cell CLL/lymphoma 3	19q13.32	44742621	44760044	**PCAT19**
**BCL6**	B-cell CLL/lymphoma 6	3q27.3	187721377	187745725	
**BCL9**	B-cell CLL/lymphoma 9	1q21.2	147541394	147626219	**RP11-14N7.2; RP11-94I2.4; RP11-196G18.22; RP4-790G17.7**
**BDNF**	brain derived neurotrophic factor	11p14.1	27654893	27722058	
**BGLAP**	bone gamma-carboxyglutamate protein	1q22	156241962	156243332	**ASH1L-AS1; RP11-404O13.5**
**BIK**	BCL2 interacting killer	22q13.2	43110748	43129712	
**BIRC2**	baculoviral IAP repeat containing 2	11q22.2	102347182	102378670	
**BIRC5**	baculoviral IAP repeat containing 5	17q25.3	78214196	78225635	
**BMI1**	BMI1 proto-oncogene, polycomb ring finger	10p12.2	22321210	22331485	
**BMP2**	bone morphogenetic protein 2	20p12.3	6767700	6780280	
**BMP4**	bone morphogenetic protein 4	14q22.2	53949736	53956862	**RP11-1085N6.5**
**BMP6**	bone morphogenetic protein 6	6p24.3	7726778	7881728	**RP3-512B11.3; TFAP2A-AS1**
**BNIP3**	BCL2 interacting protein 3	10q26.3	131967683	131982013	**RP11-432J24.2**
**BRAF**	B-Raf proto-oncogene, serine/threonine kinase	7q34	140719331	140924764	**AC078842.3**
**BSG**	basigin (Ok blood group)	19p13.3	571277	583493	
**BTK**	Bruton tyrosine kinase	Xq22.1	101349447	101390796	
**BTRC**	beta-transducin repeat containing E3 ubiquitin protein ligase	10q24.32	101353808	101557321	
**BUB1**	BUB1 mitotic checkpoint serine/threonine kinase	2q13	110637698	110678107	
**C1orf35**	chromosome 1 open reading frame 35	1q42.13	228100727	228103462	
**CALCR**	calcitonin receptor	7q21.3	93424487	93574730	**DLX6-AS1**

**CARD11**	caspase recruitment domain family member 11	7p22.2	2906075	3043945	**AC226118.1; RP11-1246C19.1; ELFN1-AS1**
**CASP10**	caspase 10	2q33.1	201182881	201229406	
**CASP3**	caspase 3	4q35.1	184627696	184649475	**AC108142.1**
**CASP8**	caspase 8	2q33.1	201233443	201287711	
**CASR**	calcium sensing receptor	3q13.33-q2	122183683	122286503	**RP11-9N20.3**
**CBX7**	chromobox 7	22q13.1	39130772	39152661	
**CCHCR1**	coiled-coil alpha-helical rod protein 1	6p21.33	31142439	31158238	**XXbac-BPG308K3.5; TRIM31-AS1**
**CCL14**	C-C motif chemokine ligand 14	17q12	35983656	35986728	**AC084809.2; CTB-58E17.1**
**CCL2**	C-C motif chemokine ligand 2	17q12	34255277	34257203	**RP11-848P1.5; AC084809.2**
**CCL20**	C-C motif chemokine ligand 20	2q36.3	227813842	227817564	**RP11-314B1.2; AC010149.4**
**CCL25**	C-C motif chemokine ligand 25	19p13.2	8052318	8062663	**CTD-3214H19.6**
**CCL3**	C-C motif chemokine ligand 3	17q12	36088256	36090160	**AC084809.2; CTB-58E17.1**
**CCL4**	C-C motif chemokine ligand 4	17q12	36103827	36105621	**AC084809.2; CTB-58E17.1**
**CCNB1**	cyclin B1	5q13.2	69167010	69178245	**CTD-2631K10.1**
**CCND1**	cyclin D1	11q13.3	69641105	69654474	**RP11-169D4.2**
**CCND2**	cyclin D2	12p13.32	4273736	4305356	**CACNA1C-AS2; CACNA1C-AS1; RP5-1063M23.2; CCND2-AS1; SCARNA10**
**CCNE1**	cyclin E1	19q12	29811994	29824317	**AC005780.1; AC093074.1; AC005307.1; AC005307.4; AC005616.1; AC005394.1; AC079466.1**
**CCNK**	cyclin K	14q32.2	99481402	99511515	**RP11-433J8.1**
**CCR2**	C-C motif chemokine receptor 2	3p21.31	46353744	46360940	**RP4-555D20.4; RP4-555D20.2; LARS2-AS1; CELSR3-AS1**
**CCR5**	C-C motif chemokine receptor 5 (gene/pseudogene)	3p21.31	46370142	46376206	**RP4-555D20.4; RP4-555D20.2; LARS2-AS1; CELSR3-AS1**
**CCR6**	C-C motif chemokine receptor 6	6q27	167111807	167139141	
**CD163**	CD163 molecule	12p13.31	7470816	7503818	**CCND2-AS1; SCARNA10; RP11-90D4.3**

**CD19**	CD19 molecule	16p11.2	28931735	28939347	**RP11-297C4.1; RP11-452L6.7**
**CD200**	CD200 molecule	3q13.2	112332224	112362812	
**CD226**	CD226 molecule	18q22.2	69860228	69962086	
**CD244**	CD244 molecule	1q23.3	160830160	160862902	**RP11-404O13.5; RP11-404F10.2**
**CD274**	CD274 molecule	9p24.1	5450503	5470567	
**CD28**	CD28 molecule	2q33.2	203706475	203739756	**AC007879.5**
**CD34**	CD34 molecule	1q32.2	207886538	207911338	
**CD38**	CD38 molecule	4p15.32	15778265	15853243	
**CD3EAP**	CD3e molecule associated protein	19q13.32	45406129	45410766	**PCAT19**
**CD4**	CD4 molecule	12p13.31	6789472	6820810	**RP5-1063M23.2; CCND2-AS1; SCARNA10; RP11-90D4.3**
**CD40**	CD40 molecule	20q13.12	46118242	46129745	
**CD40LG**	CD40 ligand	Xq26.3	136648177	136660390	**MIR503HG**
**CD44**	CD44 molecule (Indian blood group)	11p13	35138870	35232402	
**CD46**	CD46 molecule	1q32.2	207752038	207795516	
**CD47**	CD47 molecule	3q13.12	108043094	108094200	**LINC00882; LINC01215**
**CD74**	CD74 molecule	5q33.1	150401637	150412936	
**CD79A**	CD79a molecule	19q13.2	41877120	41881372	**PCAT19**
**CD79B**	CD79b molecule	17q23.3	63928738	63932344	
**CD80**	CD80 molecule	3q13.33	119523909	119559709	
**CD81**	CD81 molecule	11p15.5	2376177	2397419	**RP11-326C3.2**
**CD82**	CD82 molecule	11p11.2	44565591	44620363	**DKFZp779M0652**
**CD8A**	CD8a molecule	2p11.2	86784605	86808396	
**CD9**	CD9 molecule	12p13.31	6199844	6238271	**CACNA1C-AS2; CACNA1C-AS1; RP5-1063M23.2; CCND2-AS1; SCARNA10; RP11-90D4.3**
**CDC20**	cell division cycle 20	1p34.2	43358955	43363203	**CYP4A22-AS1**
**CDC37**	cell division cycle 37	19p13.2	10391128	10403595	**CTD-3214H19.6**
**CDCA7L**	cell division cycle associated 7 like	7p15.3	21900899	21945924	
**CDH12**	cadherin 12	5p14.3	21750191	22853622	
**CDH13**	cadherin 13	16q23.3	82626794	83796610	**RP11-679B19.1; RP11-70D24.2; LINC01229; RP11-568J23.8**
**CDH2**	cadherin 2	18q12.1	27932878	28177446	
**CDH6**	cadherin 6	5p13.3	31193655	31329146	
**CDK2**	cyclin dependent kinase 2	12q13.2	55966769	55972789	
**CDK5**	cyclin dependent kinase 5	7q36.1	151053812	151057965	

**CDKN1A**	cyclin dependent kinase inhibitor 1A	6p21.2	36676460	36687339	
**CDKN1B**	cyclin dependent kinase inhibitor 1B	12p13.1	12717270	12722383	
**CDKN2A**	cyclin dependent kinase inhibitor 2A	9p21.3	21967752	21995043	
**CDKN2B**	cyclin dependent kinase inhibitor 2B	9p21.3	22002903	22009313	
**CDKN2B-AS**	CDKN2B antisense RNA 1	9p21.3	21994791	22121097	**RP11-80I3.1**
**CDKN2C**	cyclin dependent kinase inhibitor 2C	1p32.3	50968695	50974637	**CYP4A22-AS1; RP11-191G24.1**
**CDKN3**	cyclin dependent kinase inhibitor 3	14q22.2	54396939	54420218	**RP11-1085N6.5; CTD-2002H8.2**
**CDSN**	corneodesmosin	6p21.33	31115088	31120475	**XXbac-BPG308K3.5; TRIM31-AS1**
**CEACAM6**	carcinoembryonic antigen related cell adhesion molecule 6	19q13.2	41755421	41772211	**PCAT19**
**CEBPB**	CCAAT/enhancer binding protein beta	20q13.13	50190583	50192690	**AC005220.3**
**CFLAR**	CASP8 and FADD like apoptosis regulator	2q33.1	201116015	201172688	
**CHI3L1**	chitinase 3 like 1	1q32.1	203178931	203186794	**RP11-92G12.3; PCAT6**
**CIAPIN1**	cytokine induced apoptosis inhibitor 1	16q21	57428169	57447528	**RP11-212I21.4; RP11-461O7.1**
**CIITA**	class II major histocompatibility complex transactivator	16p13.13	10866208	10941562	**RP11-490O6.2; CTD-2583P5.3**
**CIP2A**	cell proliferation regulating inhibitor of protein phosphatase 2A	3q13.13	108545752	108589644	**LINC00882; LINC01215**
**CKS1B**	CDC28 protein kinase regulatory subunit 1B	1q21.3	154974642	154979249	**ASH1L-AS1; RP11-404O13.5**
**CMTM5**	CKLF like MARVEL transmembrane domain containing 5	14q11.2	23376439	23379772	**RPPH1**
**CNMD**	chondromodulin	13q14.3	52703264	52739812	
**COL18A1**	collagen type XVIII alpha 1 chain	21q22.3	45405137	45513720	**KB-68A7.1; AP001065.15; TSPEAR-AS1**
**COL1A1**	collagen type I alpha 1 chain	17q21.33	50184096	50201648	**CTD-2377D24.6**
**COPS9**	COP9 signalosome subunit 9	2q37.3	240126548	240136802	
**CPB2**	carboxypeptidase B2	13q14.13	46053186	46105076	**RP11-5G9.5**
**CR2**	complement C3d receptor 2	1q32.2	207454300	207489895	
**CRBN**	cereblon	3p26.2	3148490	3179717	**CNTN4-AS1**
**CRP**	C-reactive protein	1q23.2	159712289	159714623	**RP11-404O13.5; RP11-404F10.2**
**CSNK1A1**	casein kinase 1 alpha 1	5q32	149495894	149551552	
**CSNK2A1**	casein kinase 2 alpha 1	20p13	482694	543838	
**CST3**	cystatin C	20p11.21	23627897	23638048	**RP5-965G21.4**

**CTAG1B**	cancer/testis antigen 1B	Xq28	154617609	154619271	
**CTAG2**	cancer/testis antigen 2	Xq28	154651972	154653579	
**CTLA4**	cytotoxic T-lymphocyte associated protein 4	2q33.2	203867788	203873960	**AC007879.5**
**CTNNB1**	catenin beta 1	3p22.1	41199451	41240448	**RP4-555D20.4; RP4-555D20.2**
**CTSK**	cathepsin K	1q21.3	150796208	150808441	**RP11-94I2.4; RP11-196G18.22; RP4-790G17.7**
**CUL4A**	cullin 4A	13q34	113208193	113265078	**GAS6-AS1; GAS6-AS2**
**CX3CL1**	C-X3-C motif chemokine ligand 1	16q21	57372461	57385048	**RP11-212I21.4; RP11-461O7.1**
**CX3CR1**	C-X3-C motif chemokine receptor 1	3p22.2	39263494	39281735	
**CXCL10**	C-X-C motif chemokine ligand 10	4q21.1	76021116	76023536	
**CXCL12**	C-X-C motif chemokine ligand 12	10q11.21	44292088	44385097	
**CXCL8**	C-X-C motif chemokine ligand 8	4q13.3	73740506	73743716	**RP11-46J23.1**
**CXCR4**	C-X-C motif chemokine receptor 4	2q22.1	136114349	136118155	
**CYLD**	CYLD lysine 63 deubiquitinase	16q12.1	50742026	50801935	
**CYP1B1**	cytochrome P450 family 1 subfamily B member 1	2p22.2	38067603	38076181	**SOS1-IT1**
**CYP2C19**	cytochrome P450 family 2 subfamily C member 19	10q23.33	94762681	94853205	
**CYP2C8**	cytochrome P450 family 2 subfamily C member 8	10q23.33	95036772	95069512	
**CYP2D6**	cytochrome P450 family 2 subfamily D member 6	22q13.2	42125531	42130881	
**CYP3A4**	cytochrome P450 family 3 subfamily A member 4	7q22.1	99756960	99784188	**DLX6-AS1; CTB-107G13.1**
**CYP3A5**	cytochrome P450 family 3 subfamily A member 5	7q22.1	99648189	99680026	**DLX6-AS1; CTB-107G13.1**
**CYR61**	cysteine rich angiogenic inducer 61	1p22.3	85580761	85583967	**LINC01140; FLJ27354**
**DAZAP2**	DAZ associated protein 2	12q13.13	51238724	51246717	
**DCC**	DCC netrin 1 receptor	18q21.2	52340172	53535903	
**DDX5**	DEAD-box helicase 5	17q23.3	64498254	64507038	**RP11-147L13.8**
**DEPDC1**	DEP domain containing 1	1p31.3	68474152	68497221	
**DEPTOR**	DEP domain containing MTOR interacting protein	8q24.12	119873655	120056202	**KB-1471A8.1**
**DIABLO**	diablo IAP-binding mitochondrial protein	12q24.31	122207662	122227534	**RP11-340F14.6; RP11-347I19.7; LINC00939**
**DICER1**	dicer 1, ribonuclease III	14q32.13	95086228	95158010	**RP11-433J8.1**
**DIO1**	iodothyronine deiodinase 1	1p32.3	53894187	53911086	**RP11-191G24.1**
**DIS3**	DIS3 homolog, exosome endoribonuclease and 3'-5' exoribonuclease	13q21.33	72755402	72782128	

**DKK1**	dickkopf WNT signaling pathway inhibitor 1	10q21.1	52314281	52317657	**RP11-96B5.3; PRKG1-AS1**
**DLL1**	delta like canonical Notch ligand 1	6q27	170282200	170291075	
**DNAH11**	dynein axonemal heavy chain 11	7p15.3	21543215	21901568	
**DNMT1**	DNA methyltransferase 1	19p13.2	10133344	10195135	**CTD-3214H19.6**
**DNMT3A**	DNA methyltransferase 3 alpha	2p23.3	25232961	25342590	
**DNMT3B**	DNA methyltransferase 3 beta	20q11.21	32762385	32809356	
**DTNB**	dystrobrevin beta	2p23.3	25377220	25673647	
**DTX3L**	deltex E3 ubiquitin ligase 3L	3q21.1	122564338	122575203	**RP11-9N20.3**
**EEF1A2**	eukaryotic translation elongation factor 1 alpha 2	20q13.33	63488012	63499315	**C20orf197; RP11-93B14.9**
**EGFR**	epidermal growth factor receptor	7p11.2	55019032	55207338	
**EGR1**	early growth response 1	5q31.2	138465492	138469315	
**EIF2S1**	eukaryotic translation initiation factor 2 subunit alpha	14q23.3	67360317	67386516	
**EIF4E**	eukaryotic translation initiation factor 4E	4q23	98878456	98930635	
**EIF4G1**	eukaryotic translation initiation factor 4 gamma 1	3q27.1	184314495	184335358	
**ELL2**	elongation factor for RNA polymerase II 2	5q15	95885098	95962288	**CTD-2154I11.2**
**ENG**	endoglin	9q34.11	127815012	127854773	**SLC25A25-AS1; RP11-339B21.10**
**EP300**	E1A binding protein p300	22q13.2	41092610	41180077	
**EPAS1**	endothelial PAS domain protein 1	2p21	46297402	46386703	
**EPHA4**	EPH receptor A4	2q36.1	221418027	221574202	
**EPHB2**	EPH receptor B2	1p36.12	22710770	22921500	
**EPHB4**	EPH receptor B4	7q22.1	100802565	100827521	**DLX6-AS1; CTB-107G13.1**
**EPHX1**	epoxide hydrolase 1	1q42.12	225810074	225845563	
**EPO**	erythropoietin	7q22.1	100720800	100723700	**DLX6-AS1; CTB-107G13.1**
**EPOR**	erythropoietin receptor	19p13.2	11377205	11384342	**CTD-3214H19.6**
**ERBB3**	erb-b2 receptor tyrosine kinase 3	12q13.2	56080025	56103507	
**ERN1**	endoplasmic reticulum to nucleus signaling 1	17q23.3	64039142	64130819	
**ESM1**	endothelial cell specific molecule 1	5q11.2	54977867	54985586	**CTD-2081C10.1; CTD-2310F14.1**
**ESR1**	estrogen receptor 1	6q25.1-q2[illegible]	151654148	152103274	
**EZH2**	enhancer of zeste 2 polycomb repressive complex 2 subunit	7q36.1	148807372	148884662	
**F11R**	F11 receptor	1q23.3	160995211	161021343	**RP11-404O13.5; RP11-404F10.2**
**FAIM**	Fas apoptotic inhibitory molecule	3q22.3	138608163	138633376	
**FAM46C**	family with sequence similarity 46 member C	1p12	117605982	117628389	**HIPK1-AS1**
**FASLG**	Fas ligand	1q24.3	172659008	172666873	**RP11-160H22.5**

**FBXW7**	F-box and WD repeat domain containing 7	4q31.3	152321258	152536095	**RP11-424M21.1; RP11-73G16.2; RP11-588K22.2**
**FCGR2B**	Fc fragment of IgG receptor IIb	1q23.3	161647243	161678654	**RP11-404O13.5; RP11-404F10.2**
**FCGR3B**	Fc fragment of IgG receptor IIIb	1q23.3	161623196	161631963	**RP11-404O13.5; RP11-404F10.2**
**FCRL4**	Fc receptor like 4	1q23.1	157573749	157598080	**ASH1L-AS1; RP11-404O13.5; RP11-404F10.2**
**FGF2**	fibroblast growth factor 2	4q28.1	122826708	122898235	
**FGF23**	fibroblast growth factor 23	12p13.32	4368227	4379728	**CACNA1C-AS2; CACNA1C-AS1; RP5-1063M23.2; CCND2-AS1; SCARNA10; RP11-90D4.3**
**FGFR3**	fibroblast growth factor receptor 3	4p16.3	1793299	1808872	**RP11-572O17.1**
**FLT1**	fms related tyrosine kinase 1	13q12.3	28300346	28495128	
**FLT4**	fms related tyrosine kinase 4	5q35.3	180601506	180650298	
**FN1**	fibronectin 1	2q35	215360440	215436167	
**FOXM1**	forkhead box M1	12p13.33	2857681	2877155	**RP11-598F7.3; CACNA1C-AS2; CACNA1C-AS1; RP5-1063M23.2; CCND2-AS1; SCARNA10**
**FRZB**	frizzled related protein	2q32.1	182833275	182866770	**AC007966.1**
**FTH1**	ferritin heavy chain 1	11q12.3	61964285	61967660	
**FTL**	ferritin light chain	19q13.33	48965309	48966879	**PTOV1-AS1**
**FYN**	FYN proto-oncogene, Src family tyrosine kinase	6q21	111660332	111873452	**RP11-425D10.10**
**GAB1**	GRB2 associated binding protein 1	4q31.21	143336830	143474565	**HHIP-AS1**
**GAB2**	GRB2 associated binding protein 2	11q14.1	78215290	78417822	
**GAS6**	growth arrest specific 6	13q34	113820549	113864103	**GAS6-AS1; GAS6-AS2**
**GATA2**	GATA binding protein 2	3q21.3	128479422	128493187	
**GBA**	glucosylceramidase beta	1q22	155234448	155244862	**ASH1L-AS1; RP11-404O13.5**
**GDE1**	glycerophosphodiester phosphodiesterase 1	16p12.3	19501693	19522145	**CTD-2288F12.1; CRYM-AS1**
**GDF15**	growth differentiation factor 15	19p13.11	18386158	18389176	
**GFI1**	growth factor independent 1 transcriptional repressor	1p22.1	92473043	92486876	**FLJ27354; RP4-639F20.1**
**GPR37**	G protein-coupled receptor 37	7q31.33	124745601	124766025	
**GPRC5D**	G protein-coupled receptor class C group 5 member D	12p13.1	12940518	12955908	

**GRK6**	G protein-coupled receptor kinase 6	5q35.3	177426496	177442849	
**GRM3**	glutamate metabotropic receptor 3	7q21.11-q2	86643914	86864879	
**GSK3A**	glycogen synthase kinase 3 alpha	19q13.2	42230186	42243330	**PCAT19**
**GSK3B**	glycogen synthase kinase 3 beta	3q13.33	119821321	120094417	**RP11-9N20.3**
**GSTM1**	glutathione S-transferase mu 1	1p13.3	109687796	109693745	**RP11-356N1.2; SLC16A1-AS1**
**GSTP1**	glutathione S-transferase pi 1	11q13.2	67583595	67586653	
**H2AFX**	H2A histone family member X	11q23.3	119093874	119095467	**USP2-AS1**
**HAMP**	hepcidin antimicrobial peptide	19q13.12	35282346	35285143	**CTC-526N19.1; CTC-523E23.1; CTC-523E23.11; TMEM147-AS1**
**HAS1**	hyaluronan synthase 1	19q13.41	51713112	51723992	**PTOV1-AS1; AC008440.10**
**HAVCR2**	hepatitis A virus cellular receptor 2	5q33.3	157085832	157109237	**AC008697.1**
**HCK**	HCK proto-oncogene, Src family tyrosine kinase	20q11.21	32052188	32101856	
**HDAC1**	histone deacetylase 1	1p35.2-p35	32292103	32333628	**MATN1-AS1**
**HDAC4**	histone deacetylase 4	2q37.3	239048168	239401647	
**HERC2**	HECT and RLD domain containing E3 ubiquitin protein ligase 2	15q13.1	28111037	28322173	**RP11-345J18.2**
**HGF**	hepatocyte growth factor	7q21.11	81699006	81770438	**AC006145.4**
**HGFAC**	HGF activator	4p16.3	3441933	3449495	**RP11-572O17.1**
**HIF1A**	hypoxia inducible factor 1 alpha subunit	14q23.2	61695401	61748259	**CTD-2002H8.2**
**HIST2H3C**	histone cluster 2 H3 family member c	1q21.2	149840687	149841193	**RP11-94I2.4; RP11-196G18.22; RP4-790G17.7**
**HK2**	hexokinase 2	2p12	74832655	74893354	**DGUOK-AS1; BOLA3-AS1**
**HLA-A**	major histocompatibility complex, class I, A	6p22.1	29942470	29945884	**XXbac-BPG308K3.5; TRIM31-AS1**
**HLA-B**	major histocompatibility complex, class I, B	6p21.33	31353866	31357245	**XXbac-BPG308K3.5; TRIM31-AS1**
**HLA-DRB1**	major histocompatibility complex, class II, DR beta 1	6p21.32	32578769	32589836	**XXbac-BPG308K3.5; TRIM31-AS1**
**HLA-G**	major histocompatibility complex, class I, G	6p22.1	29826967	29831130	**XXbac-BPG308K3.5; TRIM31-AS1**
**HMMR**	hyaluronan mediated motility receptor	5q34	163460511	163491945	
**HMOX1**	heme oxygenase 1	22q12.3	35381067	35394214	
**HNF1A**	HNF1 homeobox A	12q24.31	120978515	121002512	**RP11-340F14.6; RP11-347I19.7**

**HOMER1**	homer scaffolding protein 1	5q14.1	79373824	79514568	**CTD-2015H6.3**
**HOXB7**	homeobox B7	17q21.32	48607232	48611021	**CTD-2377D24.6**
**HPSE**	heparanase	4q21.23	83292461	83335153	
**HRAS**	HRas proto-oncogene, GTPase	11p15.5	532242	535567	**RP11-326C3.2**
**HSF1**	heat shock transcription factor 1	8q24.3	144291588	144314722	**SMPD5**
**HSP90AA1**	heat shock protein 90 alpha family class A member 1	14q32.31	102080738	102139749	**RP11-731F5.2; KIAA0125**
**HSPA1A**	heat shock protein family A (Hsp70) member 1A	6p21.33	31815514	31817942	**XXbac-BPG308K3.5; TRIM31-AS1**
**HSPA5**	heat shock protein family A (Hsp70) member 5	9q33.3	125234848	125241387	**MIR600HG; SLC25A25-AS1; RP11-339B21.10**
**HSPA8**	heat shock protein family A (Hsp70) member 8	11q24.1	123057492	123062366	**USP2-AS1**
**HSPB1**	heat shock protein family B (small) member 1	7q11.23	76302558	76304297	
**HSPB8**	heat shock protein family B (small) member 8	12q24.23	119178790	119194746	**RP11-340F14.6; RP11-347I19.7**
**IBSP**	integrin binding sialoprotein	4q22.1	87799550	87812449	
**ICAM1**	intercellular adhesion molecule 1	19p13.2	10270841	10286615	**CTD-3214H19.6**
**ID4**	inhibitor of DNA binding 4, HLH protein	6p22.3	19837370	19842200	
**IDO1**	indoleamine 2,3-dioxygenase 1	8p11.21	39913809	39928790	**RP11-350N15.3; RP11-350N15.6**
**IER3**	immediate early response 3	6p21.33	30743199	30744550	**XXbac-BPG308K3.5; TRIM31-AS1**
**IFI6**	interferon alpha inducible protein 6	1p35.3	27666061	27672213	**MATN1-AS1**
**IFNA2**	interferon alpha 2	9p21.3	21384255	21385397	
**IFNAR1**	interferon alpha and beta receptor subunit 1	21q22.11	33324443	33360361	**URB1-AS1**
**IFNAR2**	interferon alpha and beta receptor subunit 2	21q22.11	33229895	33264513	**URB1-AS1**
**IFNG**	interferon gamma	12q15	68154770	68159741	**LINC01481**
**IFNL1**	interferon lambda 1	19q13.2	39296325	39298672	**TMEM147-AS1; PCAT19**
**IGF1**	insulin like growth factor 1	12q23.2	102395867	102481839	
**IGF1R**	insulin like growth factor 1 receptor	15q26.3	98648539	98964530	**NR2F2-AS1; RP11-35O15.1; SPATA41**
**IGFBP1**	insulin like growth factor binding protein 1	7p12.3	45888360	45893668	**LINC00957**
**IGFBP2**	insulin like growth factor binding protein 2	2q35	216632828	216664436	
**IGFBP3**	insulin like growth factor binding protein 3	7p12.3	45912245	45921272	**LINC00957**
**IGFBP7**	insulin like growth factor binding protein 7	4q12	57031071	57110385	

**IGH**	immunoglobulin heavy locus	14q32.33	105586437	106879844	**RP11-731F5.2; KIAA0125**
**IGHM**	immunoglobulin heavy constant mu	14q32.33	105851966	105856217	**RP11-731F5.2; KIAA0125**
**IGK**	immunoglobulin kappa locus	2p11.2	88857361	90235368	
**IGL**	immunoglobulin lambda locus	22q11.22	22026076	22922913	**MIAT**
**IGLC2**	immunoglobulin lambda constant 2	22q11.22	22900976	22901295	**MIAT**
**IKBKB**	inhibitor of nuclear factor kappa B kinase subunit beta	8p11.21	42271302	42332653	**RP11-350N15.3; RP11-350N15.6**
**IKBKG**	inhibitor of nuclear factor kappa B kinase subunit gamma	Xq28	154542240	154565046	
**IKZF1**	IKAROS family zinc finger 1	7p12.2	50303453	50405101	
**IL10**	interleukin 10	1q32.1	206767603	206772494	**PCAT6**
**IL11**	interleukin 11	19q13.42	55364382	55370463	**AC008440.10**
**IL11RA**	interleukin 11 receptor subunit alpha	9p13.3	34652185	34661902	**EBLN3; FAM95C; FAM201A**
**IL12RB2**	interleukin 12 receptor subunit beta 2	1p31.3	67307351	67397730	
**IL16**	interleukin 16	15q25.1	81181844	81312763	
**IL17A**	interleukin 17A	6p12.2	52186387	52190638	
**IL17RA**	interleukin 17 receptor A	22q11.1	17084959	17115694	**LL22NC03-N14H11.1; AC005301.9**
**IL18**	interleukin 18	11q23.1	112143251	112164117	**RP11-629G13.1**
**IL1A**	interleukin 1 alpha	2q14.1	112773915	112785398	**DPP10-AS1**
**IL1B**	interleukin 1 beta	2q14.1	112829758	112836842	**DPP10-AS1**
**IL1RN**	interleukin 1 receptor antagonist	2q14.1	113099365	113134016	**DPP10-AS1**
**IL2**	interleukin 2	4q27	122449479	122456495	
**IL20**	interleukin 20	1q32.1	206865247	206869223	
**IL21**	interleukin 21	4q27	122612628	122621057	
**IL21R**	interleukin 21 receptor	16p12.1	27402162	27452045	**RP11-297C4.1**
**IL27**	interleukin 27	16p12.1-p1	28499362	28526730	**RP11-297C4.1; RP11-452L6.7**
**IL3**	interleukin 3	5q31.1	132060654	132063203	
**IL33**	interleukin 33	9p24.1	6214591	6257983	
**IL37**	interleukin 37	2q14.1	112908889	112918882	**DPP10-AS1**
**IL4**	interleukin 4	5q31.1	132673986	132682678	
**IL6**	interleukin 6	7p15.3	22725889	22732002	
**IL6R**	interleukin 6 receptor	1q21.3	154405193	154469450	**ASH1L-AS1; RP11-404O13.5**
**ILF2**	interleukin enhancer binding factor 2	1q21.3	153661788	153671028	**RP11-196G18.22; RP4-790G17.7; ASH1L-AS1**
**ILK**	integrin linked kinase	11p15.4	6603708	6610874	**CTD-2516F10.2**
**INHBA**	inhibin beta A subunit	7p14.1	41685101	41710532	**LINC00957**
**IQGAP1**	IQ motif containing GTPase activating protein 1	15q26.1	90388241	90502243	**RP11-648K4.2; LINC00930**
**IRAK1**	interleukin 1 receptor associated kinase 1	Xq28	154010506	154019906	

**IRF4**	interferon regulatory factor 4	6p25.3	391739	411443	**SERPINB9P1; LINC01011**
**IRF8**	interferon regulatory factor 8	16q24.1	85899168	85922606	**RP11-568J23.8**
**IRS1**	insulin receptor substrate 1	2q36.3	226731317	226798790	**RP11-314B1.2; AC010149.4**
**ITGA4**	integrin subunit alpha 4	2q31.3	181456892	181538928	
**ITGA5**	integrin subunit alpha 5	12q13.13	54395261	54419266	
**ITGAV**	integrin subunit alpha V	2q32.1	186590063	186680902	**AC007966.1**
**ITGB1**	integrin subunit beta 1	10p11.22	32900318	32958365	
**ITGB3**	integrin subunit beta 3	17q21.32	47253842	47312711	**CTD-2377D24.6**
**JAG1**	jagged 1	20p12.2	10637684	10674046	**RP4-742J24.2**
**JAG2**	jagged 2	14q32.33	105140981	105168824	**RP11-731F5.2; KIAA0125**
**JAK1**	Janus kinase 1	1p31.3	64833223	65067746	
**JAK2**	Janus kinase 2	9p24.1	4985086	5128183	
**JUN**	Jun proto-oncogene, AP-1 transcription factor subunit	1p32.1	58780791	58784113	
**JUNB**	JunB proto-oncogene, AP-1 transcription factor subunit	19p13.13	12791496	12793311	
**KCNH2**	potassium voltage-gated channel subfamily H member 2	7q36.1	150944956	150978314	
**KCNRG**	potassium channel regulator	13q14.2	50015254	50020922	
**KDM3A**	lysine demethylase 3A	2p11.2	86440647	86492716	
**KDR**	kinase insert domain receptor	4q12	55078259	55125595	
**KIT**	KIT proto-oncogene receptor tyrosine kinase	4q12	54657928	54740715	
**KLF2**	Kruppel like factor 2	19p13.11	16324826	16328662	
**KLF4**	Kruppel like factor 4	9q31.2	107484852	107489720	
**KLF9**	Kruppel like factor 9	9q21.12	70384597	70414657	**BANCR; RP11-548B3.3**
**KLRK1**	killer cell lectin like receptor K1	12p13.2	10372353	10390054	**SCARNA10; RP11-90D4.3**
**KPNB1**	karyopherin subunit beta 1	17q21.32	47649838	47683638	**CTD-2377D24.6**
**KRAS**	KRAS proto-oncogene, GTPase	12p12.1	25204789	25252093	
**KREMEN1**	kringle containing transmembrane protein 1	22q12.1	29073078	29168333	**MIAT; PIK3IP1-AS1**
**KREMEN2**	kringle containing transmembrane protein 2	16p13.3	2964216	2968383	**RP11-161M6.2; AC005606.14; RP11-304L19.12; CTD-3126B10.1; RP11-473M20.16**
**KRT81**	keratin 81	12q13.13	52285913	52291515	
**LAG3**	lymphocyte activating 3	12p13.31	6772483	6778455	**RP5-1063M23.2; CCND2-AS1; SCARNA10; RP11-90D4.3**
**LAMP2**	lysosomal associated membrane protein 2	Xq24	120426148	120469349	**RHOXF1-AS1**
**LCN2**	lipocalin 2	9q34.11	128149430	128153455	**SLC25A25-AS1; RP11-339B21.10**

**LCOR**	ligand dependent nuclear receptor corepressor	10q24.1	96832250	96964441	
**LIG4**	DNA ligase 4	13q33.3	108207442	108218368	**RP11-272L14.2**
**LILRB1**	leukocyte immunoglobulin like receptor B1	19q13.42	54616930	54637925	**AC008440.10**
**LINC02269**	long intergenic non-protein coding RNA 2269	4q34.1	173896716	173929525	
**LOC10028**	COP9 signalosome subunit 9 pseudogene	14q22.1	52798707	52799115	
**LRP1**	LDL receptor related protein 1	12q13.3	57128401	57213377	
**LRP5**	LDL receptor related protein 5	11q13.2	68298866	68449275	
**LRP6**	LDL receptor related protein 6	12p13.2	12116025	12267021	**RP11-90D4.3**
**LTA**	lymphotoxin alpha	6p21.33	31560550	31574324	**XXbac-BPG308K3.5; TRIM31-AS1**
**LY9**	lymphocyte antigen 9	1q23.3	160796074	160828256	**RP11-404O13.5; RP11-404F10.2**
**LYN**	LYN proto-oncogene, Src family tyrosine kinase	8q12.1	55877305	56012447	
**MAF**	MAF bZIP transcription factor	16q23.2	79202624	79600725	**RP11-679B19.1; RP11-70D24.2; LINC01229**
**MAFB**	MAF bZIP transcription factor B	20q12	40685848	40689240	
**MAGEA3**	MAGE family member A3	Xq28	152698742	152702347	
**MAGEC1**	MAGE family member C1	Xq27.2	141903856	141909401	
**MAGEC2**	MAGE family member C2	Xq27.2	142202342	142205290	
**MALAT1**	metastasis associated lung adenocarcinoma transcript 1 (non-protein coding)	11q13.1	65497679	65504494	
**MAP3K8**	mitogen-activated protein kinase kinase kinase 8	10p11.23	30434021	30461833	
**MAPK1**	mitogen-activated protein kinase 1	22q11.22	21759657	21867680	
**MAPK3**	mitogen-activated protein kinase 3	16p11.2	30114105	30123309	**RP11-297C4.1; RP11-452L6.7**
**MAPK7**	mitogen-activated protein kinase 7	17p11.2	19377721	19383544	
**MAPK8**	mitogen-activated protein kinase 8	10q11.22	48306639	48439360	**RP11-96B5.3; PRKG1-AS1**
**MAPK9**	mitogen-activated protein kinase 9	5q35.3	180233594	180292071	
**MAPKAPK**	mitogen-activated protein kinase-activated protein kinase 2	1q32.1	206684912	206734285	**PCAT6**
**MARCKS**	myristoylated alanine rich protein kinase C substrate	6q21	113857335	113863475	
**MBL2**	mannose binding lectin 2	10q21.1	52764977	52772845	**RP11-96B5.3; PRKG1-AS1**
**MCL1**	MCL1, BCL2 family apoptosis regulator	1q21.2	150574551	150579738	**RP11-94I2.4; RP11-196G18.22; RP4-790G17.7**

**MDK**	midkine	11p11.2	46380784	46383837	**DKFZp779M0652**
**MDM2**	MDM2 proto-oncogene	12q15	68808149	68845544	**LINC01481**
**MECOM**	MDS1 and EVI1 complex locus	3q26.2	169083499	169663781	
**MED24**	mediator complex subunit 24	17q21.1	40019097	40054636	**CTB-58E17.1; RARA-AS1; RAMP2-AS1**
**MEFV**	MEFV, pyrin innate immunity regulator	16p13.3	3242028	3256776	**RP11-161M6.2; AC005606.14; RP11-304L19.12; CTD-3126B10.1; RP11-473M20.16**
**MEG3**	maternally expressed 3 (non-protein coding)	14q32.2	100826108	100861026	
**MERTK**	MER proto-oncogene, tyrosine kinase	2q13	111898479	112039946	**DPP10-AS1**
**MET**	MET proto-oncogene, receptor tyrosine kinase	7q31.2	116672359	116798386	**ST7-AS1**
**MIB2**	mindbomb E3 ubiquitin protein ligase 2	1p36.33	1615188	1630610	
**MICA**	MHC class I polypeptide-related sequence A	6p21.33	31399784	31415315	**XXbac-BPG308K3.5; TRIM31-AS1**
**MICB**	MHC class I polypeptide-related sequence B	6p21.33	31494881	31511124	**XXbac-BPG308K3.5; TRIM31-AS1**
**MIR125A**	microRNA 125a	19q13.41	51693254	51693339	**PTOV1-AS1; AC008440.10**
**MIR125B1**	microRNA 125b-1	11q24.1	122099757	122099844	**USP2-AS1**
**MIR125B2**	microRNA 125b-2	21q21.1	16590237	16590325	**AF127936.3**
**MIR126**	microRNA 126	9q34.3	136670602	136670686	**CCDC183-AS1**
**MIR1271**	microRNA 1271	5q35.2	176367946	176368031	
**MIR130A**	microRNA 130a	11q12.1	57641198	57641286	
**MIR135B**	microRNA 135b	1q32.1	205448302	205448398	**PCAT6**
**MIR137**	microRNA 137	1p21.3	98046070	98046171	**RP4-639F20.1**
**MIR140**	microRNA 140	16q22.1	69933081	69933180	**RP11-96D1.11**
**MIR145**	microRNA 145	5q32	149430646	149430733	
**MIR146A**	microRNA 146a	5q33.3	160485352	160485450	**AC008697.1**
**MIR148A**	microRNA 148a	7p15.2	25949919	25949986	**HOXA-AS2**
**MIR155**	microRNA 155	21q21.3	25573980	25574044	**LINC00158**
**MIR15A**	microRNA 15a	13q14.2	50049119	50049201	
**MIR16-1**	microRNA 16-1	13q14.2	50048973	50049061	
**MIR17HG**	miR-17-92a-1 cluster host gene	13q31.3	91347820	91354575	
**MIR181A1**	microRNA 181a-1	1q32.1	198859044	198859153	**RP11-92G12.3; PCAT6**
**MIR18A**	microRNA 18a	13q31.3	91350751	91350821	
**MIR197**	microRNA 197	1p13.3	109598893	109598967	**RP11-356N1.2; SLC16A1-AS1**
**MIR199A1**	microRNA 199a-1	19p13.2	10817426	10817496	**CTD-3214H19.6**
**MIR199A2**	microRNA 199a-2	1q24.3	172144535	172144644	**RP11-160H22.5**
**MIR202**	microRNA 202	10q26.3	133247511	133247620	**RP11-432J24.2**

**MIR203A**	microRNA 203a	14q32.33	104117405	104117514	**RP11-731F5.2; KIAA0125**
**MIR20A**	microRNA 20a	13q31.3	91351065	91351135	
**MIR21**	microRNA 21	17q23.1	59841266	59841337	**RP11-670E13.6**
**MIR221**	microRNA 221	Xp11.3	45746157	45746266	**MIR222HG; RP6-99M1.3**
**MIR222**	microRNA 222	Xp11.3	45747015	45747124	**MIR222HG; RP6-99M1.3**
**MIR223**	microRNA 223	Xq12	66018870	66018979	
**MIR23B**	microRNA 23b	9q22.32	95085208	95085304	
**MIR29A**	microRNA 29a	7q32.3	130876747	130876810	**RP11-775D22.2**
**MIR29B1**	microRNA 29b-1	7q32.3	130877459	130877539	**RP11-775D22.2**
**MIR29B2**	microRNA 29b-2	1q32.2	207802443	207802523	
**MIR301A**	microRNA 301a	17q22	59151136	59151221	**RP11-670E13.6**
**MIR30A**	microRNA 30a	6q13	71403551	71403621	**KCNQ5-IT1**
**MIR330**	microRNA 330	19q13.32	45638994	45639087	
**MIR34B**	microRNA 34b	11q23.1	111512938	111513021	**RP11-629G13.1**
**MIR34C**	microRNA 34c	11q23.1	111513439	111513515	**RP11-629G13.1**
**MIR375**	microRNA 375	2q35	219001645	219001708	
**MIR451A**	microRNA 451a	17q11.2	28861369	28861440	**AC010761.8; RP11-848P1.5**
**MIR483**	microRNA 483	11p15.5	2134134	2134209	**RP11-326C3.2**
**MIR485**	microRNA 485	14q32.31	101055419	101055491	
**MIR631**	microRNA 631	15q24.2	75353611	75353685	**RP11-1006G14.1; LOXL1-AS1**
**MIR9-1**	microRNA 9-1	1q22	156420341	156420429	**ASH1L-AS1; RP11-404O13.5**
**MIR9-3**	microRNA 9-3	15q26.1	89368017	89368106	**RP11-648K4.2; LINC00930**
**MIR99A**	microRNA 99a	21q21.1	16539089	16539169	**AF127936.3**
**MIRLET7B**	microRNA let-7b	22q13.31	46113686	46113768	**CTA-280A3.2**
**MKI67**	marker of proliferation Ki-67	10q26.2	128096659	128126405	
**MKNK1**	MAP kinase interacting serine/threonine kinase 1	1p33	46557407	46607140	**CYP4A22-AS1**
**MMP1**	matrix metallopeptidase 1	11q22.2	102789910	102798235	
**MMP13**	matrix metallopeptidase 13	11q22.2	102942992	102955734	
**MMP2**	matrix metallopeptidase 2	16q12.2	55478830	55506691	**RP11-212I21.4; RP11-461O7.1**
**MMP9**	matrix metallopeptidase 9	20q13.12	46008908	46016561	
**MRC1**	mannose receptor C-type 1	10p12.33	17809343	17911162	**ST8SIA6-AS1**
**MS4A1**	membrane spanning 4-domains A1	11q12.2	60455809	60470752	
**MSLN**	mesothelin	16p13.3	760746	768865	**RP11-161M6.2; AC005606.14; RP11-304L19.12; CTD-3126B10.1; RP11-473M20.16**
**MTDH**	metadherin	8q22.1	97643972	97730260	**KB-1958F4.1; KB-1460A1.5**

**MTHFR**	methylenetetrahydrofolate reductase	1p36.22	11785730	11806103	
**MTOR**	mechanistic target of rapamycin kinase	1p36.22	11106531	11262551	
**MTRR**	5-methyltetrahydrofolate-homocysteine methyltransferase reductase	5p15.31	7869104	7901124	
**MUC1**	mucin 1, cell surface associated	1q22	155185824	155192915	**ASH1L-AS1; RP11-404O13.5**
**MUC16**	mucin 16, cell surface associated	19p13.2	8848840	9010390	**CTD-3214H19.6**
**MVP**	major vault protein	16p11.2	29820394	29848039	**RP11-297C4.1; RP11-452L6.7**
**MXI1**	MAX interactor 1, dimerization protein	10q25.2	110207605	110287365	
**MYC**	MYC proto-oncogene, bHLH transcription factor	8q24.21	127736069	127741434	
**MYD88**	myeloid differentiation primary response 88	3p22.2	38138478	38143022	
**MYEOV**	myeloma overexpressed	11q13.3	69294138	69297287	**RP11-169D4.2**
**MYNN**	myoneurin	3q26.2	169772247	169789716	
**NAMPT**	nicotinamide phosphoribosyltransferase	7q22.3	106248285	106285732	**CTB-107G13.1**
**NBEA**	neurobeachin	13q13.3	34942287	35672737	
**NCAM1**	neural cell adhesion molecule 1	11q23.2	112961247	113278436	**RP11-629G13.1**
**NCOR2**	nuclear receptor corepressor 2	12q24.31	124324411	124567464	**RP11-340F14.6; RP11-347I19.7; LINC00939**
**NCR1**	natural cytotoxicity triggering receptor 1	19q13.42	54906042	54938211	**AC008440.10**
**NCR3**	natural cytotoxicity triggering receptor 3	6p21.33	31588883	31593024	**XXbac-BPG308K3.5; TRIM31-AS1**
**NEK2**	NIMA related kinase 2	1q32.3	211658256	211675630	
**NES**	nestin	1q23.1	156668763	156677397	**ASH1L-AS1; RP11-404O13.5; RP11-404F10.2**
**NFIL3**	nuclear factor, interleukin 3 regulated	9q22.31	91409045	91425063	**RP11-367F23.1**
**NFKB1**	nuclear factor kappa B subunit 1	4q24	102501329	102617302	
**NFKB2**	nuclear factor kappa B subunit 2	10q24.32	102394110	102402529	
**NFKBIA**	NFKB inhibitor alpha	14q13.2	35401510	35404754	
**NOS1AP**	nitric oxide synthase 1 adaptor protein	1q23.3	162069791	162370023	**RP11-404O13.5; RP11-404F10.2**
**NOTCH1**	notch 1	9q34.3	136494433	136545786	**CCDC183-AS1**
**NOTCH2**	notch 2	1p12	119911553	120073449	
**NPM1**	nucleophosmin 1	5q35.1	171387648	171410900	
**NQO1**	NAD(P)H quinone dehydrogenase 1	16q22.1	69709401	69726668	**RP11-96D1.11**
**NR1I2**	nuclear receptor subfamily 1 group I member 2	3q13.33	119780484	119818485	**RP11-9N20.3**

**NR1I3**	nuclear receptor subfamily 1 group I member 3	1q23.3	161229666	161238623	**RP11-404O13.5; RP11-404F10.2**
**NRAS**	NRAS proto-oncogene, GTPase	1p13.2	114704464	114716894	**SLC16A1-AS1; HIPK1-AS1**
**NSD1**	nuclear receptor binding SET domain protein 1	5q35.3	177133079	177300213	
**NSD2**	nuclear receptor binding SET domain protein 2	4p16.3	1871357	1982207	**RP11-572O17.1**
**NSD3**	nuclear receptor binding SET domain protein 3	8p11.23	38275042	38382272	**RP11-350N15.3; RP11-350N15.6**
**NTRK2**	neurotrophic receptor tyrosine kinase 2	9q21.33	84668368	85027070	
**ORC4**	origin recognition complex subunit 4	2q23.1	147930397	148021604	
**OSM**	oncostatin M	22q12.2	30262828	30266843	**MIAT; PIK3IP1-AS1**
**P2RX7**	purinergic receptor P2X 7	12q24.31	121132819	121189478	**RP11-340F14.6; RP11-347I19.7**
**PAK1**	p21 (RAC1) activated kinase 1	11q13.5-q1	77322015	77474063	
**PAK2**	p21 (RAC1) activated kinase 2	3q29	196739857	196832647	**XXYLT1-AS2; LMLN-AS1**
**PAK4**	p21 (RAC1) activated kinase 4	19q13.2	39125780	39179406	**TMEM147-AS1; PCAT19**
**PARP14**	poly(ADP-ribose) polymerase family member 14	3q21.1	122680726	122730840	**RP11-9N20.3**
**PASD1**	PAS domain containing repressor 1	Xq28	151563535	151676739	
**PAX5**	paired box 5	9p13.2	36833274	37035949	**EBLN3; FAM95C; FAM201A**
**PCDH10**	protocadherin 10	4q28.3	133149290	133208606	
**PCNA**	proliferating cell nuclear antigen	20p12.3	5114953	5126622	
**PDCD1**	programmed cell death 1	2q37.3	241849881	241858908	
**PDCD5**	programmed cell death 5	19q13.11	32581161	32587452	**AC005394.1; AC079466.1; CTC-526N19.1; CTC-523E23.1; CTC-523E23.11; TMEM147-AS1**
**PDGFA**	platelet derived growth factor subunit A	7p22.3	497245	520668	**AC226118.1; RP11-1246C19.1; ELFN1-AS1**
**PDGFB**	platelet derived growth factor subunit B	22q13.1	39223359	39245055	
**PDIA2**	protein disulfide isomerase family A member 2	16p13.3	283118	287209	**RP11-161M6.2; AC005606.14; RP11-304L19.12; CTD-3126B10.1; RP11-473M20.16**

**PDPK1**	3-phosphoinositide dependent protein kinase 1	16p13.3	2537964	2603190	**RP11-161M6.2; AC005606.14; RP11-304L19.12; CTD-3126B10.1; RP11-473M20.16**
**PDZK1**	PDZ domain containing 1	1q21.1	145670852	145707507	**RP11-14N7.2; RP11-94I2.4**
**PF4**	platelet factor 4	4q13.3	73980825	73982124	**RP11-46J23.1**
**PIAS3**	protein inhibitor of activated STAT 3	1q21.1	145848522	145859081	**RP11-14N7.2; RP11-94I2.4; RP11-196G18.22**
**PIK3CA**	phosphatidylinositol-4,5-bisphosphate 3-kinase catalytic subunit alpha	3q26.32	179148114	179240093	
**PIK3CB**	phosphatidylinositol-4,5-bisphosphate 3-kinase catalytic subunit beta	3q22.3	138652698	138834938	
**PIK3CG**	phosphatidylinositol-4,5-bisphosphate 3-kinase catalytic subunit gamma	7q22.3	106865278	106908978	**CTB-107G13.1**
**PIK3R1**	phosphoinositide-3-kinase regulatory subunit 1	5q13.1	68215737	68301821	
**PIK3R2**	phosphoinositide-3-kinase regulatory subunit 2	19p13.11	18153178	18170533	
**PIM2**	Pim-2 proto-oncogene, serine/threonine kinase	Xp11.23	48913182	48919136	**MIR222HG; RP6-99M1.3**
**PKM**	pyruvate kinase M1/2	15q23	72199029	72231624	**RP11-1006G14.1; LOXL1-AS1**
**PLAT**	plasminogen activator, tissue type	8p11.21	42174718	42207724	**RP11-350N15.3; RP11-350N15.6**
**PLAU**	plasminogen activator, urokinase	10q22.2	73909182	73917501	**DNAJC9-AS1; RP11-399K21.14**
**PLAUR**	plasminogen activator, urokinase receptor	19q13.31	43646095	43670346	**PCAT19**
**PLK1**	polo like kinase 1	16p12.2	23678772	23690367	**CRYM-AS1**
**PLK2**	polo like kinase 2	5q11.2	58453982	58460139	**CTD-2310F14.1**
**PMAIP1**	phorbol-12-myristate-13-acetate-induced protein 1	18q21.32	59899960	59904306	**RP11-108P20.4; RP11-299P2.2**
**PML**	promyelocytic leukemia	15q24.1	73994673	74047819	**RP11-1006G14.1; LOXL1-AS1**
**POLQ**	DNA polymerase theta	3q13.33	121431420	121546006	**RP11-9N20.3**
**PON1**	paraoxonase 1	7q21.3	95298357	95324572	**DLX6-AS1**
**POU5F1**	POU class 5 homeobox 1	6p21.33	31164337	31170693	**XXbac-BPG308K3.5; TRIM31-AS1**
**PPARG**	peroxisome proliferator activated receptor gamma	3p25.2	12287850	12471054	
**PPARGC1A**	PPARG coactivator 1 alpha	4p15.2	23792021	24472975	
**PPIA**	peptidylprolyl isomerase A	7p13	44796636	44803123	**LINC00957**

**PPP2R2B**	protein phosphatase 2 regulatory subunit Bbeta	5q32	146589504	147081520	
**PRDM1**	PR/SET domain 1	6q21	106046729	106109939	**RP11-425D10.10**
**PRKAB1**	protein kinase AMP-activated non-catalytic subunit beta 1	12q24.23	119667864	119681624	**RP11-340F14.6; RP11-347I19.7**
**PROCR**	protein C receptor	20q11.22	35171937	35215989	
**PROK1**	prokineticin 1	1p13.3	110451166	110457354	**RP11-356N1.2; SLC16A1-AS1; HIPK1-AS1**
**PROKR1**	prokineticin receptor 1	2p13.3	68645822	68655576	**AC007392.3**
**PSMA6**	proteasome subunit alpha 6	14q13.2	35278558	35317479	
**PSMB5**	proteasome subunit beta 5	14q11.2	23016543	23035220	**RPPH1**
**PSMB9**	proteasome subunit beta 9	6p21.32	32854161	32859851	**TRIM31-AS1**
**PSORS1C1**	psoriasis susceptibility 1 candidate 1	6p21.33	31114831	31140092	**XXbac-BPG308K3.5; TRIM31-AS1**
**PSORS1C2**	psoriasis susceptibility 1 candidate 2	6p21.33	31137534	31139350	**XXbac-BPG308K3.5; TRIM31-AS1**
**PTBP1**	polypyrimidine tract binding protein 1	19p13.3	797392	812327	
**PTEN**	phosphatase and tensin homolog	10q23.31	87863438	87971930	
**PTGS2**	prostaglandin-endoperoxide synthase 2	1q31.1	186671812	186680427	**GS1-115G20.1**
**PTK2**	protein tyrosine kinase 2	8q24.3	140658382	141001313	**SMPD5**
**PTK2B**	protein tyrosine kinase 2 beta	8p21.2	27311482	27459391	**RP11-51J9.5**
**PTN**	pleiotrophin	7q33	137227341	137343865	**AC078842.3**
**PTPN6**	protein tyrosine phosphatase, non-receptor type 6	12p13.31	6946576	6961316	**RP5-1063M23.2; CCND2-AS1; SCARNA10; RP11-90D4.3**
**PTPRC**	protein tyrosine phosphatase, receptor type C	1q31.3-q32	198638968	198757476	**RP11-92G12.3**
**PTTG1**	pituitary tumor-transforming 1	5q33.3	160421807	160428744	**AC008697.1**
**PTX3**	pentraxin 3	3q25.32	157436791	157443628	
**PVR**	poliovirus receptor	19q13.31	44643798	44666161	**PCAT19**
**RAC1**	Rac family small GTPase 1	7p22.1	6374495	6403967	**AC007009.1; AC007128.1**
**RAD23B**	RAD23 homolog B, nucleotide excision repair protein	9q31.2	107283236	107332194	
**RAF1**	Raf-1 proto-oncogene, serine/threonine kinase	3p25.2	12583601	12664201	
**RANBP2**	RAN binding protein 2	2q13	108719446	109842301	
**RARA**	retinoic acid receptor alpha	17q21.2	40309171	40357643	**CTB-58E17.1; RARA-AS1; RAMP2-AS1**
**RASD1**	ras related dexamethasone induced 1	17p11.2	17494437	17496395	
**RASSF1**	Ras association domain family member 1	3p21.31	50329786	50340936	**CELSR3-AS1**

**RB1**	RB transcriptional corepressor 1	13q14.2	48303747	48481890	
**RBBP5**	RB binding protein 5, histone lysine methyltransferase complex subunit	1q32.1	205086142	205122022	**PCAT6**
**RECQL**	RecQ like helicase	12p12.1	21468910	21501669	
**RELA**	RELA proto-oncogene, NF-kB subunit	11q13.1	65653596	65662972	
**RELB**	RELB proto-oncogene, NF-kB subunit	19q13.32	45001449	45038198	**PCAT19**
**RELN**	reelin	7q22.1	103471784	103989516	**CTB-107G13.1**
**RGS1**	regulator of G protein signaling 1	1q31.2	192575727	192580029	**RP5-1011O1.2**
**RHOA**	ras homolog family member A	3p21.31	49359136	49412097	**LARS2-AS1; CELSR3-AS1**
**RHOD**	ras homolog family member D	11q13.2	67056818	67072017	
**ROR2**	receptor tyrosine kinase like orphan receptor 2	9q22.31	91722596	91950206	**RP11-367F23.1**
**RPSA**	ribosomal protein SA	3p22.1	39406689	39412542	
**RUNX1**	runt related transcription factor 1	21q22.12	34787801	35049334	**URB1-AS1**
**RUNX2**	runt related transcription factor 2	6p21.1	45328142	45664032	
**S1PR1**	sphingosine-1-phosphate receptor 1	1p21.2	101236749	101241520	
**SCARA3**	scavenger receptor class A member 3	8p21.1	27633665	27734027	**RP11-51J9.5**
**SDC1**	syndecan 1	2p24.1	20200797	20225433	
**SELENOW**	selenoprotein W	19q13.33	47778585	47784682	**PTOV1-AS1**
**SELP**	selectin P	1q24.2	169588849	169630139	
**SELPLG**	selectin P ligand	12q24.11	108621895	108633894	
**SEMG1**	semenogelin I	20q13.12	45206964	45209773	
**SERPINE1**	serpin family E member 1	7q22.1	101127087	101139266	**CTB-107G13.1**
**SERPINF1**	serpin family F member 1	17p13.3	1761965	1777565	**AC108004.3**
**SFRP1**	secreted frizzled related protein 1	8p11.21	41261957	41309471	**RP11-350N15.3; RP11-350N15.6**
**SFRP2**	secreted frizzled related protein 2	4q31.3	153780590	153789076	**RP11-424M21.1; RP11-73G16.2; RP11-588K22.2**
**SFRP4**	secreted frizzled related protein 4	7p14.1	37905932	37916923	
**SFRP5**	secreted frizzled related protein 5	10q24.2	97766751	97771999	
**SGK1**	serum/glucocorticoid regulated kinase 1	6q23.2	134169246	134318112	**LINC01013; TARID**
**SGK3**	serum/glucocorticoid regulated kinase family member 3	8q13.1	66712418	66862022	**LINC01289; LACTB2-AS1**
**SH3GL1**	SH3 domain containing GRB2 like 1, endophilin A2	19p13.3	4360367	4400568	**CTD-3214H19.6**
**SHC3**	SHC adaptor protein 3	9q22.1	89005771	89182308	**RP11-367F23.1**
**SHH**	sonic hedgehog	7q36.3	155799984	155812273	**AC073133.1**
**SIRT1**	sirtuin 1	10q21.3	67884669	67918390	**LINC01515; RP11-343J3.2**
**SIRT6**	sirtuin 6	19p13.3	4174109	4182604	**CTD-3214H19.6**

**SKP2**	S-phase kinase associated protein 2	5p13.2	36151989	36192792	**GDNF-AS1**
**SLAMF7**	SLAM family member 7	1q23.3	160739057	160754821	**RP11-404O13.5; RP11-404F10.2**
**SLC40A1**	solute carrier family 40 member 1	2q32.2	189560590	189580811	**AC007966.1**
**SLC7A5**	solute carrier family 7 member 5	16q24.2	87830022	87869499	**RP11-568J23.8**
**SMAD2**	SMAD family member 2	18q21.1	47833095	47931193	**RP11-456K23.1**
**SMO**	smoothened, frizzled class receptor	7q32.1	129188872	129213548	**RP11-775D22.2**
**SNAI1**	snail family transcriptional repressor 1	20q13.13	49982976	49988886	**AC005220.3**
**SNORD25**	small nucleolar RNA, C/D box 25	11q12.3	62855565	62855631	
**SNORD27**	small nucleolar RNA, C/D box 27	11q12.3	62855012	62855083	
**SNORD30**	small nucleolar RNA, C/D box 30	11q12.3	62853663	62853732	
**SOCS1**	suppressor of cytokine signaling 1	16p13.13	11254417	11256182	**RP11-490O6.2; CTD-2583P5.3**
**SOCS3**	suppressor of cytokine signaling 3	17q25.3	78356777	78360079	**RP13-516M14.1**
**SOD1**	superoxide dismutase 1	21q22.11	31659622	31668931	**URB1-AS1**
**SOD2**	superoxide dismutase 2	6q25.3	159679064	159762529	
**SOST**	sclerostin	17q21.31	43753731	43758788	**RARA-AS1; RAMP2-AS1**
**SOX4**	SRY-box 4	6p22.3	21593741	21598619	
**SP1**	Sp1 transcription factor	12q13.13	53380195	53416446	
**SP7**	Sp7 transcription factor	12q13.13	53326575	53344793	
**SPA17**	sperm autoantigenic protein 17	11q24.2	124673844	124694794	**RP11-744N12.3**
**SPACA3**	sperm acrosome associated 3	17q11.2	32991864	32997877	**RP11-848P1.5; AC084809.2**
**SPANXB1**	SPANX family member B1	Xq27.1	141002591	141003706	
**SPDYA**	speedy/RINGO cell cycle regulator family member A	2p23.2	28810834	28850610	
**SPI1**	Spi-1 proto-oncogene	11p11.2	47354858	47395640	**DKFZp779M0652**
**SPP1**	secreted phosphoprotein 1	4q22.1	87975650	87983411	
**SRGN**	serglycin	10q22.1	69057544	69104811	**LINC01515; RP11-343J3.2**
**SRI**	sorcin	7q21.12	88205115	88226993	
**SSX1**	SSX family member 1	Xp11.23	48255317	48267444	**MIR222HG; RP6-99M1.3**
**SSX2**	SSX family member 2	Xp11.22	52696896	52707227	
**SSX4**	SSX family member 4	Xp11.23	48383527	48393343	**MIR222HG; RP6-99M1.3**
**ST3GAL6**	ST3 beta-galactoside alpha-2,3-sialyltransferase 6	3q12.1	98732236	98795845	
**STAT1**	signal transducer and activator of transcription 1	2q32.2	190969036	191014250	
**STAT3**	signal transducer and activator of transcription 3	17q21.2	42313324	42388505	**CTB-58E17.1; RARA-AS1; RAMP2-AS1**

**STAT5A**	signal transducer and activator of transcription 5A	17q21.2	42287547	42311943	**CTB-58E17.1; RARA-AS1; RAMP2-AS1**
**STOML2**	stomatin like 2	9p13.3	35099776	35103195	**EBLN3; FAM95C; FAM201A**
**SULF1**	sulfatase 1	8q13.2-q13	69466624	69660912	**LACTB2-AS1**
**SULF2**	sulfatase 2	20q13.12	47654584	47786629	
**TANK**	TRAF family member associated NFKB activator	2q24.2	161136955	161236176	
**TAP1**	transporter 1, ATP binding cassette subfamily B member	6p21.32	32845209	32853971	**TRIM31-AS1**
**TAP2**	transporter 2, ATP binding cassette subfamily B member	6p21.32	32821833	32838823	**XXbac-BPG308K3.5; TRIM31-AS1**
**TBXA2R**	thromboxane A2 receptor	19p13.3	3594506	3608749	
**TCF19**	transcription factor 19	6p21.33	31158524	31164215	**XXbac-BPG308K3.5; TRIM31-AS1**
**TDG**	thymine DNA glycosylase	12q23.3	103965815	103988878	
**TERC**	telomerase RNA component	3q26.2	169764610	169765060	
**TERF1**	telomeric repeat binding factor 1	8q21.11	73008862	73047752	**LACTB2-AS1**
**TERF2**	telomeric repeat binding factor 2	16q22.1	69355561	69386004	**RP11-96D1.11**
**TERT**	telomerase reverse transcriptase	5p15.33	1253167	1295626	
**TGFB1**	transforming growth factor beta 1	19q13.2	41330531	41353933	**PCAT19**
**TGFBR2**	transforming growth factor beta receptor 2	3p24.1	30606490	30694142	
**TGFBR3**	transforming growth factor beta receptor 3	1p22.1	91680343	91906002	**FLJ27354; RP4-639F20.1**
**THBS1**	thrombospondin 1	15q14	39581079	39598918	**RP11-128A17.1; RP11-23P13.6**
**THPO**	thrombopoietin	3q27.1	184371935	184379688	
**TIMP1**	TIMP metallopeptidase inhibitor 1	Xp11.3	47582291	47586791	**MIR222HG; RP6-99M1.3**
**TIMP2**	TIMP metallopeptidase inhibitor 2	17q25.3	78852977	78925390	**RP13-516M14.1**
**TIRAP**	TIR domain containing adaptor protein	11q24.2	126281268	126294933	**RP11-744N12.3**
**TJP1**	tight junction protein 1	15q13.1	29699367	29968919	**RP11-345J18.2; RP11-3D4.4**
**TLR3**	toll like receptor 3	4q35.1	186069155	186086724	
**TLR4**	toll like receptor 4	9q33.1	117704175	117717491	**RP11-9M16.2**
**TLR5**	toll like receptor 5	1q41	223108401	223143282	
**TNF**	tumor necrosis factor	6p21.33	31575567	31578336	**XXbac-BPG308K3.5; TRIM31-AS1**
**TNFAIP3**	TNF alpha induced protein 3	6q23.3	137866317	137883314	
**TNFRSF10A**	TNF receptor superfamily member 10a	8p21.3	23191457	23225167	
**TNFRSF10B**	TNF receptor superfamily member 10b	8p21.3	23020133	23069187	

**TNFRSF11A**	TNF receptor superfamily member 11a	18q21.33	62325287	62388096	**RP11-108P20.4; RP11-299P2.2; RP11-793J2.1**
**TNFRSF11E**	TNF receptor superfamily member 11b	8q24.12	118923557	118952144	**KB-1471A8.1**
**TNFRSF13E**	TNF receptor superfamily member 13B	17p11.2	16939084	16972088	
**TNFRSF13C**	TNF receptor superfamily member 13C	22q13.2	41925032	41926817	
**TNFRSF17**	TNF receptor superfamily member 17	16p13.13	11965107	11968068	**RP11-490O6.2; CTD-2583P5.3**
**TNFRSF1A**	TNF receptor superfamily member 1A	12p13.31	6328757	6342117	**CACNA1C-AS2; CACNA1C-AS1; RP5-1063M23.2; CCND2-AS1; SCARNA10; RP11-90D4.3**
**TNFRSF1B**	TNF receptor superfamily member 1B	1p36.22	12166948	12209222	
**TNFRSF6B**	TNF receptor superfamily member 6b	20q13.33	63696651	63698698	**C20orf197; RP11-93B14.9**
**TNFRSF8**	TNF receptor superfamily member 8	1p36.22	12063318	12144213	
**TNFSF10**	TNF superfamily member 10	3q26.31	172505508	172523507	
**TNFSF11**	TNF superfamily member 11	13q14.11	42562736	42608013	**RP11-5G9.5**
**TNFSF13**	TNF superfamily member 13	17p13.1	7558292	7561608	
**TNFSF13B**	TNF superfamily member 13b	13q33.3	108268240	108308484	**RP11-272L14.2**
**TNFSF8**	TNF superfamily member 8	9q32-q33.1	114893343	114930595	**RP11-9M16.2**
**TNFSF9**	TNF superfamily member 9	19p13.3	6530999	6535928	**CTD-3214H19.6**
**TNKS**	tankyrase	8p23.1	9555229	9782346	
**TOM1**	target of myb1 membrane trafficking protein	22q12.3	35299275	35347994	
**TP53**	tumor protein p53	17p13.1	7668402	7687550	
**TP53RK**	TP53 regulating kinase	20q13.12	46684365	46689637	
**TP73-AS1**	TP73 antisense RNA 1	1p36.32	3735984	3747373	
**TPPP**	tubulin polymerization promoting protein	5p15.33	659862	730454	
**TRAF3**	TNF receptor associated factor 3	14q32.32	102777479	102911500	**RP11-731F5.2; KIAA0125**
**TRAF6**	TNF receptor associated factor 6	11p12	36483767	36510313	
**TRAPPC1**	trafficking protein particle complex 1	17p13.1	7930345	7931999	
**TRIAP1**	TP53 regulated inhibitor of apoptosis 1	12q24.31	120443961	120446412	**RP11-340F14.6; RP11-347I19.7**
**TRIM13**	tripartite motif containing 13	13q14.2	49997007	50018467	
**TSC1**	TSC complex subunit 1	9q34.13	132891348	132945269	**CCDC183-AS1**

**TSC2**	TSC complex subunit 2	16p13.3	2047804	2088720	**RP11-161M6.2; AC005606.14; RP11-304L19.12; CTD-3126B10.1; RP11-473M20.16**
**TSLP**	thymic stromal lymphopoietin	5q22.1	111070080	111078024	**CTD-2201G3.1**
**TSPAN7**	tetraspanin 7	Xp11.4	38561478	38688918	**AC092198.1**
**TWIST1**	twist family bHLH transcription factor 1	7p21.1	19113047	19117672	
**TXN**	thioredoxin	9q31.3	110243812	110256640	
**UBE2C**	ubiquitin conjugating enzyme E2 C	20q13.12	45812576	45816957	
**UCHL1**	ubiquitin C-terminal hydrolase L1	4p13	41256881	41268429	
**UCHL5**	ubiquitin C-terminal hydrolase L5	1q31.2	193012366	193060140	**RP5-1011O1.2**
**ULK4**	unc-51 like kinase 4	3p22.1	41246599	41963020	**RP4-555D20.4; RP4-555D20.2; LARS2-AS1**
**URI1**	URI1, prefoldin like chaperone	19q12	29923644	30016612	**AC005780.1; AC093074.1; AC005307.1; AC005307.4; AC005616.1; AC005394.1; AC079466.1; CTC-526N19.1**
**USO1**	USO1 vesicle transport factor	4q21.1	75724522	75814289	
**USP14**	ubiquitin specific peptidase 14	18p11.32	158483	213739	
**USP24**	ubiquitin specific peptidase 24	1p32.3	55066359	55215374	**RP11-191G24.1**
**USP9X**	ubiquitin specific peptidase 9, X-linked	Xp11.4	41085420	41236579	**AC092198.1**
**VCAM1**	vascular cell adhesion molecule 1	1p21.2	100719640	100739045	
**VCAN**	versican	5q14.2-q14	83471674	83582303	**CTD-2015H6.3**
**VDR**	vitamin D receptor	12q13.11	47841537	47905031	
**VEGFA**	vascular endothelial growth factor A	6p21.1	43770209	43786487	
**VEGFC**	vascular endothelial growth factor C	4q34.3	176683534	176792745	
**VHL**	von Hippel-Lindau tumor suppressor	3p25.3	10141635	10153670	
**VTCN1**	V-set domain containing T-cell activation inhibitor 1	1p13.1-p12	117143587	117210992	**HIPK1-AS1**
**WNT3A**	Wnt family member 3A	1q42.13	228007022	228067113	
**WNT5A**	Wnt family member 5A	3p14.3	55465715	55505261	
**WT1**	Wilms tumor 1	11p13	32387775	32435535	
**WWOX**	WW domain containing oxidoreductase	16q23.1-q2	78099413	79212667	**RP11-679B19.1; RP11-70D24.2; LINC01229**
**WWTR1**	WW domain containing transcription regulator 1	3q25.1	149517235	149724783	
**XAF1**	XIAP associated factor 1	17p13.1	6755411	6775647	

**XBP1**	X-box binding protein 1	22q12	28794560	28800572	**MIAT; PIK3IP1-AS1**
**XIAP**	X-linked inhibitor of apoptosis	Xq25	123859812	123913979	**RHOXF1-AS1; RP11-13E5.2**
**XPO1**	exportin 1	2p15	61477934	61538522	**RP11-373L24.1; RP11-568N6.1**
**XPO5**	exportin 5	6p21.1	43522330	43576075	
**XRCC3**	X-ray repair cross complementing 3	14q32.33	103697611	103715486	**RP11-731F5.2; KIAA0125**
**XRCC4**	X-ray repair cross complementing 4	5q14.2	83077409	83370333	**CTD-2015H6.3**
**XRCC5**	X-ray repair cross complementing 5	2q35	216109297	216206293	
**YBX1**	Y-box binding protein 1	1p34.2	42682235	42703803	
**YWHAZ**	tyrosine 3-monooxygenase/tryptophan 5-monooxygenase activation protein zeta	8q22.3	100918576	100954068	**KB-1958F4.1; KB-1460A1.5**
**YY1**	YY1 transcription factor	14q32.2	100238765	100279034	**RP11-433J8.1**
**ZDHHC9**	zinc finger DHHC-type containing 9	Xq26.1	129803288	129843934	**RP11-13E5.2**
**ZKSCAN3**	zinc finger with KRAB and SCAN domains 3	6p22.1	28349913	28369177	**XXbac-BPG308K3.5; TRIM31-AS1**
